# Endophytic Fungi—Alternative Sources of Cytotoxic Compounds: A Review

**DOI:** 10.3389/fphar.2018.00309

**Published:** 2018-04-26

**Authors:** Fazilath Uzma, Chakrabhavi D. Mohan, Abeer Hashem, Narasimha M. Konappa, Shobith Rangappa, Praveen V. Kamath, Bhim P. Singh, Venkataramana Mudili, Vijai K. Gupta, Chandra N. Siddaiah, Srinivas Chowdappa, Abdulaziz A. Alqarawi, Elsayed F. Abd_Allah

**Affiliations:** ^1^Microbial Metabolite Research Laboratory, Department of Microbiology and Biotechnology, Bangalore University, Bangalore, India; ^2^Department of Studies in Molecular Biology, University of Mysore, Mysore, India; ^3^Botany and Microbiology Department, College of Science, King Saud University, Riyadh, Saudi Arabia; ^4^Department of Studies in Biotechnology, University of Mysore, Mysore, India; ^5^Adichunchanagiri Institute for Molecular Medicine, BG Nagara, Mandya, India; ^6^Molecular Microbiology and Systematics Laboratory, Department of Biotechnology, Mizoram University, Aizawl, India; ^7^Microbiology Division, DRDO-BU-Centre for Life sciences, Bharathiar University, Coimbatore, India; ^8^Department of Chemistry and Biotechnology, ERA Chair of Green Chemistry, School of Science, Tallinn University of Technology, Tallinn, Estonia; ^9^Plant Production Department, College of Food and Agricultural Sciences, King Saud University, Riyadh, Saudi Arabia

**Keywords:** bioactive metabolites, anticancer agents, natural compounds, fungal alkaloids, taxol, podophyllotoxin, camptothecin, vinca alkaloids

## Abstract

Cancer is a major cause of death worldwide, with an increasing number of cases being reported annually. The elevated rate of mortality necessitates a global challenge to explore newer sources of anticancer drugs. Recent advancements in cancer treatment involve the discovery and development of new and improved chemotherapeutics derived from natural or synthetic sources. Natural sources offer the potential of finding new structural classes with unique bioactivities for cancer therapy. Endophytic fungi represent a rich source of bioactive metabolites that can be manipulated to produce desirable novel analogs for chemotherapy. This review offers a current and integrative account of clinically used anticancer drugs such as taxol, podophyllotoxin, camptothecin, and vinca alkaloids in terms of their mechanism of action, isolation from endophytic fungi and their characterization, yield obtained, and fungal strain improvement strategies. It also covers recent literature on endophytic fungal metabolites from terrestrial, mangrove, and marine sources as potential anticancer agents and emphasizes the findings for cytotoxic bioactive compounds tested against specific cancer cell lines.

## Introduction

Human health is at constant risk due to the occurrence of different types of cancers. Cancer cells are characterized by the enormous replicative potential, apoptotic resistance, and invasive ability. The dislodging of cancer cells from the primary site may lead to the formation of cancer at the secondary site by a process of metastasis which eventually results in the death of an individual. Cancer is a major cause of death globally. An estimated 1,688,780 new cancer cases and 600,920 cancer-related deaths projected to occur in the US in 2017 (Siegel et al., 2017). In 2013, 14.9 million cancer incidences, 8.2 million cancer-related deaths were reported (Global Burden of Disease Cancer, 2015). The frequency of cancer-related mortality is increasing in developing countries at an alarming rate. Therefore, several research groups have been actively involved in the development of novel anticancer drugs throughout the globe. It is estimated that the total cost for the development of a new drug is ~$2.6 billion. The global annual expenditure on anticancer drugs is ~$100 billion and is estimated to increase to $150 billion by 2020 (Prasad et al., 2017). Many available anticancer drugs exhibit toxicity to proliferating normal cells, possess adverse effects, and are less effective against several types of cancer, which results in a need for bioactive compounds from natural products (Remesh, 2017).

Scientists have begun to comprehend that plants may be reservoirs for an infinite number of microorganisms, commonly referred as endophytes (Schulz et al., 2002; Strobel et al., 2004). Endophytes inhabit at internal plant tissue in a symbiotic association and can spend most of their life cycle within host plants. Approximately one million endophytic species are present in the plant kingdom (Fouda et al., 2015). It is noteworthy to mention that, a small number of endophytic fungi were reported to produce plant growth stimulating hormones such as gibberellic acid and indole acetic acid (Rai et al., 2014). Metabolites produced by endophytes can be influenced by the chemistry of their host plants (Kusari et al., 2012). During the period of co-evolution, some of the endophytes have developed the ability to produce biologically active compounds that are similar or identical to their host plants (Zhao et al., 2011; Jia et al., 2016). Several studies have indicated the possible likelihood of medicinal plants that host endophytic fungi capable of producing pharmacologically important natural products. It is reasonable to postulate that the medicinal properties of these plants could be due to the endophytes residing within them. This has led to an investigation of beneficial compounds related to plant-endophyte interactions (Hardoim et al., 2008). The interaction between plants and endophytes is regulated by genes of both the organisms and controlled by the environmental factors (Moricca and Ragazzi, 2008). In other words, the endophyte may encounter metabolically unfavorable condition due to host defense chemicals (Schulz et al., 1999; Easton et al., 2009). The plant and endophyte association may also modulate the production of secondary metabolites in the host plant. The secondary metabolites isolated from endophytes displayed a broad spectrum of pharmacological properties including anticancer, antiviral, antibacterial, and antifungal activity (Jalgaonwala et al., 2017). In the subsequent sections, we discuss about the anticancer compounds extracted from endophytic fungus from diverse habitats.

## Natural products as sources of novel anticancer compounds

Natural products obtained from endophytic fungi have been identified as continuing and prolific sources of anticancer agents that could have a profound impact on modern medicine for the advancement of anticancer drugs (Schulz et al., 2002; Newman and Cragg, 2007). In the modern era, the discovery of potent medicines with minimal side effects acts as an alternative to conventionally used methods of disease control and treatment (Kusari et al., 2009b; Kusari and Spiteller, 2012). In 2000, it was estimated that ~57% of compounds in clinical trials for cancer therapy are natural products and their derivatives (Demain and Vaishnav, 2011). Early reports suggest that natural bioactive compounds isolated from endophytes may serve as alternate sources for the discovery of new anticancer drugs (Joseph and Priya, 2011; Alvin et al., 2014; Xie and Zhou, 2017).

Most of the reports dealing with the cytotoxicity of metabolites from endophytic fungi utilize antiproliferative assays in the cancer cell lines and high-throughput screening that essentially allows the evaluation of several compounds together for their potential anticancer activity (Aly et al., 2008; Lei et al., 2013). Isolation of natural bioactive compounds and screening them for the pharmacological properties offer a route for the discovery of drug candidates (Salvador-Reyes and Luesch, 2015). Several endophytic fungal strains have been identified and reported to produce new compounds that are effective in anticancer assays (Stierle and Stierle, 2015). Taxol, vincristine, etoposide, irinotecan, topotecan, and vinblastine are some of that plant-derived anticancer drugs that are in clinical use for the treatment of several human cancers (Balunas and Kinghorn, 2005). The review describes the isolation of some of these compounds from endophytes and reports their cytotoxic effect in various cell lines.

## Clinically used anticancer drugs and their precursors from endophytic fungi

Although several natural compounds have been shown to possess good pharmacological and anticancer activities, they often face many challenges. The major challenges in developing natural compounds as drug candidates are identification of the right source; difficulty in extraction due to the excessive degradation by metabolic enzymes; low abundance of active principles; hindrance in large-scale production because of steric complexity high chiral centers, hydrogen bond donors/acceptors, bulky compounds, and diverse aromaticity (Singh et al., 2016). Identification of the good source of natural compounds can be achieved by random selection, folklore, codified systems of medicine, ethnopharmacology, ayurvedic classical texts, or zoopharmacognosy (Singh et al., 2016). Often, plant-derived bioactive natural compounds are of low abundance. For an instance, the total taxol content of the bark of *Taxus baccata* tree ranges between 0.064 to 8.032 g/tree, and it was also reported that a tree aged about 100 years can produce the dry bark yield of 5.74 kg (Nadeem et al., 2002). Therefore, it is essential to develop alternative strategies for the production of bioactive compounds using tissue culture, synthetic/semi-synthetic approaches, biotransformation, and use of microbes that can produce desired products in large scale. This review presents bioactive compounds isolated from plant-associated fungal strains obtained from terrestrial, mangrove, and marine habitats, which are capable of inducing cytotoxicity/apoptosis in cancer cells and thereby possess efficient anticancer activity.

### Taxol

Taxol **(1)** is the world's first billion-dollar anticancer drug and it is a highly functionalized polycyclic diterpenoid that belongs to a class of taxanes. In 1962, researchers from National Cancer Institute supported project, collected inner bark (phloem-cambial tissue) of the Pacific yew tree *(Taxus brevifolia)* and analyzed for the presence of natural bioactive compounds. Initial screening of crude extract on cancer cells revealed good cytotoxic activity. It took several years to identify and isolate paclitaxel (trade name is taxol) in its pure form from the extract. Thereafter, paclitaxel was identified as a potent antitumor agent and made its way into clinical trials. One of the biggest hurdles faced during the initial days of taxol production is the requirement of six yew trees of 100 years old to treat one cancer patient (Demain and Vaishnav, 2011). In other words, 0.01 to 0.03% is the taxol content in dry weight of phloem of the yew tree. The constraints in the availability, isolation, and synthesis of taxol made the researchers to think of alternate sources for its production. The efforts resulted in the isolation of 10-deacetyl-baccatin III **(2)** (a precursor for the synthesis of taxol) from *T. baccata* (European yew). The tree is abundant and bears high amount of 10-deacetyl-baccatin III in its needles and nowadays it is used as a precursor for the synthesis of taxol by semi-synthetic approach (Tulp and Bohlin, 2002). Eventually, FDA approved taxol for the treatment of several types of tumors including breast, ovary, and Kaposi's sarcoma. It is also claimed that taxol is the best-selling cancer drug ever manufactured (Gordon, 2011) with a market size of $1.6 billion in 2005 and its structural analog, docetaxel presented the sales of $3 billion in 2009 (Demain and Vaishnav, 2011). The efficacy and increased demand for taxol resulted in developing biotechnological approaches to prepare the drug (Kusari et al., 2014). In the present day, taxol is produced by semisynthetic approaches using 10-deacetyl-baccatin III, plant cell culture, and endophytic fungi. In a breakthrough, the *T. brevifolia* associated endophytic fungus *Taxomyces andreanae* was reported to produce taxol and related compounds (Stierle et al., 1993). This extraordinary feat led to the discovery of several new taxol-producing endophytic fungi from different host plants (Strobel et al., 1996; Strobel, 2003; Zaiyou et al., 2015). The production of paclitaxel was also identified in an angiosperm named *Corylus avellena* L which belongs to the family *Betulaceae* (Qaderi et al., 2012). In the next section, we have comprehensively discussed the mode of action of taxol in cancer cells, its endophytic fungal sources and cytotoxic ability.

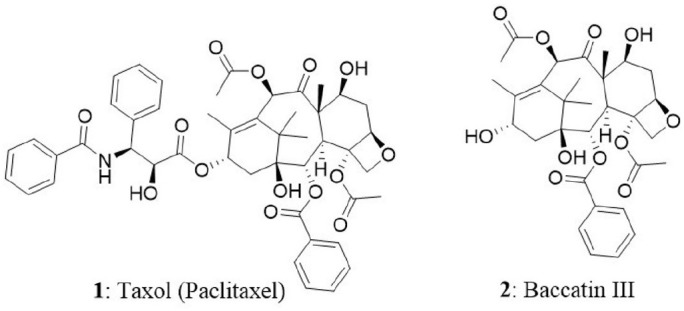



#### Mode of action

Paclitaxel represents a new class of antineoplastic agents and has a unique mode of action. It promotes and stabilizes the polymerization of microtubules and resists their depolymerization. In the presence of taxol, polymerized microtubule is resistant to depolymerization by cold (4°C) and calcium chloride (4 mM) (Manfredi et al., 1982). This unusual stability of microtubules interfere with the mitotic spindle assembly, chromosome segregation which leads to mitotic arrest and eventually cell death (Schiff et al., 1979; Horwitz et al., 1986; Weaver, 2014).

#### Endophytic fungi as producers of taxol

Several research groups have been focusing on the search for new sources of paclitaxel. Several *Taxus* species, such as *Taxus baccata, Taxus chinensis, Taxus cuspidata, Taxus x media, Taxus floridana, Taxus canadensis, Taxus yunnanensis, Taxus mairei, Taxus sumatrana*, and *Taxus wallichiana*, have been reported to produce taxol, albeit with significant variation in the taxane content within and among the different species (Tabata, 2006). Non-*Taxus* species, such as *Cardiospermum halicacabum, Citrus medica, Cupressus* sp*., Ginkgo biloba, Hibiscus rosa-sinensis, Taxodium distichum, Podocarpus* sp., *Torreya grandifolia, Terminalia arjuna*, and *Wollemia nobilis*, are also taxol producers (Flores-Bustamante et al., 2010). As discussed earlier, low taxol yields in the plants prompted researchers to discover alternate ways of taxol production which led to the identification of endophytic fungi as sources of taxol (Heinig and Jennewein, 2009). The discovery of taxol-producing endophytes from both *Taxus* and non-*Taxus* plants led to screening of array of plants for the identification of endophytes with taxol-producing abilities. Endophytic fungi belonging to the genera *Alternaria, Aspergillus, Botryodiplodia, Botrytis, Cladosporium, Ectostroma, Fusarium, Metarhizium, Monochaetia, Mucor, Ozonium, Papulaspora, Periconia, Pestalotia, Pestalotiopsis, Phyllosticta, Pithomyces*, and *Taxomyces* have been tested and reported for the production of paclitaxel and its analogs (Table 1).

**Table 1:** List of taxol-producing endophytic fungi and their host plants.

**Sl No**	**Endophytic fungus**	**Host plant**	**Taxol yield (μg/L)**	**References**
1	*Taxomyces andreanae*	*Taxus brevifolia*	0.024–0.05	Stierle et al., 1993
2	*Pestalotiopsis microspora*	*Taxus walachiana*	60–70	Strobel et al., 1996
3	*Pestalotiopsis microspora*	*Taxodium distichurn*	0.014–1.487	Li, 1996
4	*Pestalotiopsis* sp.	*Wollemia nobilis*	0.127–0.485	Strobel et al., 1997
5	*Periconia* sp.	*Torreya grandifolia*	0.030–0.831	Li, 1998
6	*Tubercularia* sp.	*Taxus mairei*	–	Wang et al., 2000
7	*Nodulisporium sylviforme*	*Taxus cuspidata*	51.06–125.7	Zhou et al., 2001
8	*Papulaspora* sp.	*Taxus chinensis var. mairei*	10.25	Hu et al., 2006
9	*Botrytis* sp.	*Taxus chinensis var. mairei*	161.24	Hu et al., 2006
10	*Fusarium maire*	*Taxus mairei*	225.2	Xu et al., 2006
11	*Ozonium* sp.	*Taxus chinensis var. mairei*	4–18	Guo et al., 2006
12	*Botryodiplodia theobromae*	*Taxus baccata*	280.5	Venkatachalam et al., 2008
13	*Pestalotiopsis pauciseta*	*Cardiospermum helicacabum*	113.3	Gangadevi et al., 2008
14	*Colletotrichum gleospoiroides*	*Justicia gendarussa*	163.4	Gangadevi and Muthumary, 2008a
15	*Bartalinia robillardoides* Tassi	*Aegle marmelos* Correa ex Roxb	187.6	Gangadevi and Muthumary, 2008b
16	*Phyllosticta citricarpa*	*Citrus medica*	137–265	Kumaran et al., 2008b
17	*Phyllosticta spinarum*	*Cupressus* sp.	125–235	Senthil Kumaran et al., 2008
18	*Phyllosticta melochiae* Yates	*Melochia Corchorifolia* L.	274	Kumaran et al., 2008a
19	*Fusarium solani*	*Taxus celebica*	1.6	Chakravarthi et al., 2008
20	*Fusarium arthrosporioides*	*Taxus cuspidata*	131	Li et al., 2008
21	*Fusarium solani*	*Taxus chinensis*	163.35	Deng et al., 2008
22	*Aspergillus fumigatus*	*Podocarpus* sp.	560	Sun et al., 2008
23	*Aspergillus niger var. taxi*	*Taxus cuspidata*	273.46	Zhao et al., 2009
24	*Phomopsis* sp. BKH 27	*Taxus cuspidata*	418	Kumaran and Hur, 2009
25	*Phomopsis* sp. BKH 30	*Gingko biloba*	372	Kumaran and Hur, 2009
26 27	*Phomopsis* sp. BKH 35	*Larix leptolepis*	334	Kumaran and Hur, 2009
28	*Phyllosticta dioscoreae*	*Hibiscus rosa-sinensis*	298	Kumaran et al., 2009
30	*Chaetomella raphigera*	*Terminalia arjuna*	79.6	Gangadevi and Muthumary, 2009a
31	*Pestalotiopsis terminaliae*	*Terminalia arjuna*	211.1	Gangadevi and Muthumary, 2009b
32	*Cladosporium cladosporioides* MD2	*Taxus media*	800	Zhang P. et al., 2009
33	*Metarhizium anisopliae*	*Taxus chinensis*	846.1	Liu K. et al., 2009
34	*Colletotrichum gloeosporioides*	*Plumeria acutifolia*	57.54	Nithya and Muthumary, 2009
35	*Paraconiothyrium* sp.	*Taxus media*	40	Soliman et al., 2011
36	*Lasiodiplodia theobromae*	*Morinda citrifolia*	245	Pandi et al., 2011
37	*Fusarium oxysporum*	*Rhizophora annamalayana*	172.3	Elavarasi et al., 2012
38	*Phoma betae*	*Ginkgo biloba*	795	Kumaran et al., 2012
39	*Fusarium solani*	*Tylophora indica*	157.38	Merlin et al., 2012
40	*Chaetomium* sp.	*Michelia champaca* L.	77.23	Rebecca et al., 2012
41	*Fusarium redolens*	*Taxus baccata L*. subsp. *wallichiana*	66	Garyali et al., 2013
42	*Penicillium aurantiogriseum NRRL 62431*	*Corylus avellana*	70–350	Yang et al., 2014
43	*Cladosporium oxysporum*	*Moringa oleifera*	550	Elavarasi et al., 2012; Gokul Raj et al., 2015
44	*Phoma* sp.	*Calotropis gigantea*	152.26	Hemamalini et al., 2015
45	*Paraconiothyrium variabile*	*Taxus baccata*	14.7	Somjaipeng et al., 2016
46	*Phoma medicaginis*	*Taxus wallichiana* var. *mairei*	1215	Zaiyou et al., 2017
47	*Aspergillus aculeatinus Tax-6*	*Taxus chinensis var. mairei*	334.92–1337.56	Qiao et al., 2017
48	*Cladosporium* sp	*Taxus baccata*	152.5 mg/Kg dry weight	Kasaei et al., 2017

Paclitaxel production from endophytes is reported often in submerged culture (Wang et al., 2001; Zaiyou et al., 2017). Paclitaxel in the fungal extract can be detected by various analytical (Thin layer chromatography, High-performance liquid chromatography), spectroscopic (Matrix-assisted laser desorption/ionization—Time of flight [MALDI-TOF], Nuclear magnetic resonance [NMR], Fast atom bombardment [FAB], Electron spray ionization [ESI]), and immunological techniques (using monoclonal antibodies specific for paclitaxel) (Flores-Bustamante et al., 2010). Polymerase chain reaction-based methods can be used for the screening and identification of taxol-producing fungi. Previous studies have revealed that presence of genes encoding for the enzymes taxadiene synthase, 10-deacetylbaccatin III-10-O-acetyltransferase, and baccatin III 13-O-(3-amino-3-phenylpropanoyl) transferase could serve as molecular markers for the identification of fungi with taxol-producing abilities (Zhang et al., 2008; Vasundhara et al., 2016). Most taxol production has been reported using a liquid media such as potato dextrose broth (PDB), modified liquid medium (MlD), and S7. However, the highest taxol yield of 846.1 μg/L was reported from the endophytic fungus, *Metarhizium anisopliae* of *T. chinensis* (Liu K. et al., 2009). The endophyte *Cladosporium cladosporioides* from *Taxus media* was also shown to produce 800 μg/L of taxol (Zhang P. et al., 2009). Sun et al. isolated 155 endophytic fungi from the tissue of Podocarpus and identified strain A2 as a taxol producer. Subsequently, A2 was classified as *Aspergillus fumigates* and the taxol yield obtained was 0.56 mg/L when grown in liquid potato dextrose medium. The isolated taxol inhibited the growth of Vero cells as similar to commercially available taxol (Sun et al., 2008). In another report, 34 endophytic fungi were isolated from the medicinal plant *Salacia oblonga* at Kigga village, Karnataka, India, and screened for their potential to produce taxol or taxanes. The authors used genomic mining approach to identify the taxol-producing fungus. Among the isolates, seven fungi were found to possess 10-deacetylbaccatin III-10-O-acetyltransferase gene and one revealed the presence of C-13 phenylpropanoid side chain-CoA acyltransferase gene (Roopa et al., 2015). Recently, 18 fungal isolates were screened for their ability to produce taxol. *Cladosporium oxysporum* isolated from *Moringa oleifera* was identified as taxol producing fungus and the taxol yield was found to be 550 μg/L. The fungal taxol suppressed the growth of HCT15 with an IC_50_ value of 3.5 μM. The presence of gene encoding 10-deacetylbaccatin III-10-O-acetyltransferase confirmed the ability of an endophyte to produce taxol (Gokul Raj et al., 2015). Zaiyou et al. isolated 528 fungal strains from the bark of *T. wallichiana* var. *mairei* and only one strain was found to produce paclitaxel. The fungal strain was identified as *Phoma medicaginis*. The amount of paclitaxel produced when grown in whole potato dextrose broth culture, spent culture medium and dry mycelium was found to be 1.215, 0.936 mg/L, and 20 mg/kg, respectively (Zaiyou et al., 2017).

#### Cytotoxic ability of taxol and its precursors

Several cytotoxicity tests have been performed to determine the anticancer abilities of taxol and its precursors. One such interesting investigation highlighted that baccatin III (biosynthetic precursor of taxol) functions *via* a similar mechanism to taxol. Taxol and baccatin III were extracted from *Fusarium solani* and tested against HeLa, HepG2, Jurkat-JR4, OVCAR-3, T47D, Jurkat-JR16, and caspase-8-deficient Jurkat cells. Both compounds were able to inhibit cell proliferation of the above-mentioned tumor-derived cell lines with IC_50_ values between 0.005 to 0.2 μM for taxol and 2 to 5 μM for baccatin III. These results indicate that although taxol induces apoptosis in the tested cells, there were difference in sensitivity between the tumor cells toward fungal taxol and baccatin III treatment. Among these two compounds, the potent inducer of apoptosis is debatable; because baccatin III is the more active molecule inside the cells during the growth period and is less active than taxol *in vitro* studies. Conversely, taxol has benefit over baccatin III in cellular uptake, microtubule binding kinetics and interaction with other proteins (Chakravarthi et al., 2013). The endophyte *Diaporthe phaseolorum* isolated from *T. wallichiana* var. *mairei* synthesizes baccatin III. The baccatin III content in PDB and spent culture medium was found to be 0.219 and 0.193 mg/L, respectively (Zaiyou et al., 2013). A detailed description of the taxoid biosynthesis pathway is the solution to improve the supply of taxol and other important taxoids. Several genes and enzymes of the *Taxus* pathway for the taxoid biosynthesis have now been established (Heinig and Jennewein, 2009). Future studies should focus on the isolation and identification of endophytes that are capable of producing a high amount of taxol, as well as optimization of the production conditions.

### Podophyllotoxin

Podophyllotoxin **(3)**, an aryl tetralin lignan derived from both gymnosperms and angiosperms, has also been investigated as an anticancer drug (Majumder and Jha, 2009); It is widely distributed in the genera *Diphylleia, Dysosma, Juniperus*, and *Sinopodophyllum* (also called *Podophyllum*) (Li et al., 2013). Podophyllotoxin and its analogs are pharmacologically important due to their cytotoxic and antiviral activities (Gordaliza et al., 1994, 2000; Abd-Elsalam and Hashim, 2013). At present, one of the major natural sources of podophyllotoxin is *Sinopodophyllum* plants. Etoposide **(4)** and teniposide **(5)** are the semisynthetic derivatives of podophyllotoxin approved for the treatment of cancer of lungs and testicles, other solid tumors and a variety of leukemias (Kusari et al., 2009a). Etoposide was developed as an alternative to podophyllotoxin in 1966 and received FDA approval in 1983. Subsequently, enzyme-assisted asymmetric synthesis of (–)-podophyllotoxin and its C2-epimer, (–)-picropodophyllin was described (Berkowitz et al., 2000). However, they are not economically feasible due to the low yield (Chandra, 2012). The supply of podophyllotoxin derivatives from traditional sources is limited due to their low abundance in the plants. A large amount of effort in the past several years has focused on improving production from different podophyllotoxin-producing plant species. Various alternative strategies have been implemented to enhance the production of podophyllotoxin-related compounds. Plant tissue culture is one of the alternate, reliable, and sustainable strategies to produce natural compounds of the plant origin (Ochoa-Villarreal et al., 2016).

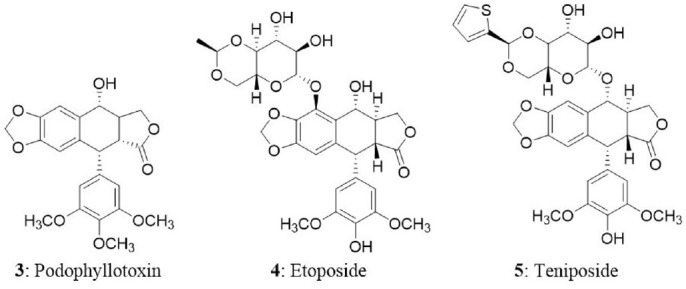



#### Mode of action

Etoposide and teniposide were reported to induce excellent antitumor activity in various cancer models. Their antitumor potential is due to their interaction with the enzyme topoisomerase II (Botta et al., 2001; Cortés and Pastor, 2003). Topoisomerase inhibitors impart their action by two mechanisms—either by eliminating the catalytic activity of topoisomerase II or by increasing the levels of topoisomerase II:DNA covalent complexes (often designated as topoisomerase II poisons) (Nitiss, 2009). Etoposide does not induce cytotoxicity by blocking the catalytic activity of topoisomerase II, but they poison mammalian topoisomerase II by dramatically increasing the covalent DNA cleavage complexes leading to the permanent double-stranded breaks. The increase in enzyme-associated DNA breaks serve as targets for genetic alterations such as recombination, exchange of sister chromatid, insertions, deletions, and translocation (Froelich-Ammon and Osheroff, 1995). The accumulation of permanent DNA breaks induces a series of events and drive the cell to death. During this process, topoisomerases serve as a physiological toxins (Van Maanen et al., 1988; Kaufmann, 1989; Hande, 1998; Pendleton et al., 2014).

#### Podophyllotoxins from endophytic fungi

The two strains of *Phialocephala fortinii* were isolated from the rhizomes of *Podophyllum peltatum* which produced podophyllotoxin and the yield ranged between 0.5 and 189 μg/L. The fungal extract was evaluated for cytotoxicity using brine shrimp lethality assay and the LD_50_ values were in the range of 2–3 μg/mL (Eyberger et al., 2006). Similarly, *Fusarium oxysporum*, an endophytic fungus isolated from *Juniperus recurva* in Gulmarg region of South Kashmir, India. The fungus was found to produce podophyllotoxin and the yield was found to be 28 μg/g of dry mass (Kour et al., 2008). In another study, the endophytic fungus *Trametes hirsute* was isolated from the dried rhizomes of *Podophyllum hexandrum* which produced podophyllotoxin, podophyllotoxin glycoside and demethoxypodophyllotoxin. The isolated metabolites exhibited cytotoxicity in U-87 cell line (Puri et al., 2006). In another study, *Aspergillus fumigatus* Fresenius was isolated from *Juniperus communis* L. Horstmann collected from botanical gardens of Rombergpark, Dortmund, Germany. The fungus was found to produce deoxypodophyllotoxin and maximum yield of 100 μg/g of dry weight of mycelia was observed (Kusari et al., 2009a). In the next study, six fungi were collected from the rhizomes of *Sinopodophyllum hexandrum* (Royle) Ying plants at Taibai Mountains of China. Among the fungi, *Mucor fragilis* Fresen. produced podophyllotoxin and kaempferol. The podophyllotoxin yield was found to be 49.3 μg/g of mycelial dry weight (Huang et al., 2014). Recently, first report was published on production of podophyllotoxin from endophyte *Alternaria tenuissima*. The fungal endophyte was isolated from fresh roots of *Sinopodophyllum emodi* (Wall.) Ying at Xinglong Mountains, Gansu Province, China. The secondary metabolite analysis revealed the presence of podophyllotoxin in the fungal biomass (Liang et al., 2016). The list of podophyllotoxin producing endophytic fungi and their host plants has been tabulated and presented as Table 2.

**Table 2:** List of podophyllotoxin producing endophytic fungi and their host plants.

**Sl No**	**Endophytic fungus**	**Host plant**	**Podophyllotoxin yield (μg/L)**	**References**
1	*Alternaria* sp.	*Podophyllum hexandrum*	–	Chandra, 2012
2	*Phialocephala fortinii*	*Podophyllum peltatum*	0.5–189	Eyberger et al., 2006
3	*Trametes hirsute*	*Podophyllum hexandrum*	30 μg/g dry weight of mycelia	Puri et al., 2006
4	*Alternaria neesex* Ty	*Sinopodophyllum hexandrum*	2.418	Cao et al., 2007
5	*Fusarium oxysporum*	*Juniperus recurva*	28 μg/g dry weight of mycelia	Kour et al., 2008
6	*Alternaria* sp.	*Sabina vulgaris*	–	Zhao et al., 2010b
7	*Alternaria* sp.	*Podophyllum hexandrum*	–	Zhao et al., 2010b
8	*Monilia* sp.	*Dysosma veitchii*	–	Zhao et al., 2010b
9	*Penicillium* sp.	*Podophyllum hexandrum*	–	Zhao et al., 2010b
10	*Penicillium* sp.	*Diphylleia sinensis*	–	Zhao et al., 2010b
11	*Fusarium solani*	*Podophyllum hexandrum*	29 μg/g dry weight of mycelia	Nadeem et al., 2012
12	*Alternaria tenuissima*	*Sinopodophyllum emodi* (Wall.) Ying	Chloroform extract: 50.5 mg; Butanol extract: 348 mg; Methanol extract of the mycelia: 1139.9 mg	Liang et al., 2016
13	*Chaetomium globosum*	*Sinopodophyllum hexandrum*	–	Wang et al., 2017
	*Pseudallescheria* sp.			

### Camptothecin

Camptothecin **(6)**, an anticancer drug that was first isolated from the bark of the *Camptotheca acuminata*. It is a pentacyclic pyrroloquinoline alkaloid and a parent compound for clinically used anticancer drugs such as irinotecan and topotecan. It is present in nature in 20-S-camptothecin form and 20-R-camptothecin enantiomer form is biologically inactive. Camptothecin is produced mainly by *C. acuminata* and *Nothapodytes foetida*. In National Cancer Institute screening studies, anticancer activity of wood extract of the *C. acuminata* was identified in 1958. In 1968, Wani and Wall isolated the cytotoxic agent from the extract of *C. acuminata* and identified the alkaloid as camptothecin (Takimoto, 2002). The purified compound exhibited good cytotoxic activity against several types of tumors. Conversely, clinical development of camptothecin was hampered in clinical trials due to poor water solubility and severe gastrointestinal toxicities (Takimoto, 2002). Eventually, water soluble and biologically active novel camptothecin derivatives were synthesized (Miyasaka et al., 1986). After four decades from the identification of antitumor activity of *C. acuminata* extract, two camptothecins, topotecan **(7)** and irinotecan **(8)** were approved by FDA for the treatment of colorectal cancer, small-cell lung cancer, and ovarian cancer (Blagosklonny, 2004). Meanwhile, camptothecin was identified to target topoisomerase-I in cells to impart its anticancer activity. Several camptothecin derivatives such as IDEC-132 (9-aminocamptothecin), rubitecan (9-nitrocamptothecin), and 10,11-methylenedioxy camptothecin have been identified as good anticancer agents (Ulukan and Swaan, 2002). In the next subsequent sections, we have discussed about the mechanism of action of camptothecin and endophytic fungi as its source.

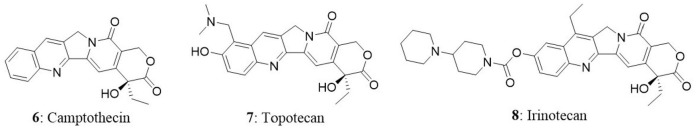



#### Mode of action

At the initial days, it was believed that camptothecins induce cytotoxicity in cancer cells by inhibiting DNA and RNA synthesis. In the late 1980s, topoisomerase I was identified as the cellular target of camptothecin. Topoisomerase-I is involved in the relaxation of DNA supercoiling that is generated as a result of replication. Topoisomerase-I creates a nick in the single strand of DNA to release supercoiling. In parallel, topoisomerase-I forms an ester linkage with the 3' end of nicked DNA through its catalytic tyrosine to form topoisomerase-I cleavage complexes. Immediately 5'hydroxyl group of the nicked DNA strand nucleophilically attacks the tyrosyl-DNA-phosphodiester bond to bring the DNA to normal topology. In general, topoisomerase-I cleavage complexes are short-lived intermediates and hard to detect in the cellular environment (Pommier, 2006). Camptothecin and its derivatives interact with topoisomerase-I cleavage complexes and stabilize them. This results in the initiation of series of apoptotic events, finally leading to cell death. Studies using mutant yeasts (*Saccharomyces cerevisiae* and *Schizosaccharomyces pombe*) that lacks topoisomerase I activity were completely resistant to camptothecin indicating the absence of off-targets (Eng et al., 1988). It is now clearly demonstrated that the antitumor activity of camptothecin is due to its ability to inhibit DNA topoisomerase- I, which is involved in the swiveling and relaxation of DNA during replication and transcription.

#### Camptothecin from endophytic fungi

Camptothecin obtained from plant sources does not meet the requirement from the global market and consistent attempts have been underway to identify novel sources of camptothecin. *Entrophospora infrequens* is the endophytic fungus isolated from the inner bark of *N. foetida* in Konkan ghats, West coast of India. The yield of camptothecin was found to be 18 μg/mg of the chloroform extract. The isolated fungal camptothecin showed good cytotoxicity against A549, HEp2, and OVCAR-5 (Puri et al., 2005). In another study, the same fungus produced 4.96 mg of camptothecin per 100 g of dry mass in 48 h in a bioreactor (Amna et al., 2006). The two endophytic fungal strains of *F. solani* were isolated from *Apodytes dimidiate* in the Western Ghats, India. In broth culture after 4 days of incubation, the yield of camptothecin produced by two strains was found to be 37 and 53 μg/100 g (Shweta et al., 2010). In another study, Shweta et al. isolated three endophytic fungi (*A. alternate, Fomitopsis* sp., *Phomopsis* sp.) from fruit and seeds of *Miquelia dentata* and found to produce camptothecin, 9-methoxycamptothecin, and 10-hydroxycamptothecin. The yield of camptothecin obtained from *A. alternata, Fomitopsis* sp., and *Phomopsis* sp. was 73.9, 55.49, and 42.06 μg/g dry weight (Shweta et al., 2013). Three camptothecin-producing fungi, *Aspergillus* sp. LY341, *Aspergillus* sp. LY355, and *Trichoderma atroviride* LY357 were isolated from *C. acuminata*. The corresponding camptothecin yields were 7.93, 42.92, and 197.82 μg/L, respectively. Unfortunately, LY341 and LY355 strains lost the camptothecin-producing capability with repetitive subculturing. On the other hand, consistent production of camptothecin was seen in LY357 from second to eighth generation (Pu et al., 2013; Kai et al., 2015).

In the next study, 161 fungi were isolated from *C. acuminata*. Among the isolates, *Botryosphaeria dothidea* X-4 fungus was reported to produce 9-methoxycamptothecin (Ding et al., 2013). The endophytic fungi *Neurospora* sp. was isolated from the seed of *N. foetida* and found to produce camptothecin. The isolated fungal camptothecin was tested against the A549 and OVCAR-5 cells and it showed good cytotoxicity in both the cell lines (Rehman et al., 2008). In another study, the fungus (XZ-01) that belongs to *Phomopsis* sp. was isolated from twigs of *C. acuminata* collected from the Jiangshi Natural Reserve, Fujian, China. Details of the camptothecin-producing fungi and their corresponding host plants are provided in Table 3.

**Table 3:** List of camptothecin, 9-methoxycamptothecin, 10-hydroxycamptothecin producing endophytic fungi, and their host plants.

**Sl No**	**Endophytic fungus**	**Host plant**	**Camptothecin yield (μg/g)**	**References**
1	*Entrophospora infrequens*	*Nothapodytes foetida*	18 μg/mg of the chloroform extract	Puri et al., 2005
2	*Entrophospora infrequens*	*Nothapodytes foetida*	49.6	Amna et al., 2006
3	*Neurospora crassa*	*Nothapodytes foetida*	–	Rehman et al., 2008
4	*Fusarium solani*	*Camptotheca acuminata*	–	Kusari et al., 2009b
5	*Fusarium solani*	*Apodytes dimidiate*	0.37	Shweta et al., 2010
6	*Fusarium solani*	*Apodytes dimidiate*	0.53	
7	*Trichoderma atroviridae*	*Camptotheca acuminata*	197.82 μg/L	Pu et al., 2013
8	*Botryosphaeriadothidea*	*Camptotheca acuminata*	–	Ding et al., 2013
9	*Fusarium nematophilum*	*Camptotheca acuminata*	37	Su et al., 2014
	*Alternaria alternata*		29	
	*Phomopsis vaccinii*		24	
	*Colletotrichum gloeosporioides*		17	
10	*Fusarium solani*	*Camptotheca acuminata*	40 μg/g	Ran et al., 2017
			150 μg/L	

### Vinca alkaloids

The success story of isolation of vinca alkaloids from Madagascar periwinkle (botanical name is *Catharanthus roseus* G. Don and commonly called *Vinca rosea*) marks back to late 1950s. In Jamaican folklore, the extracts of periwinkle were used as an oral hypoglycemic agent in the absence of insulin treatment (Noble, 1990). Eventually, two groups (Noble, Beer, and Cutts at the Collip Laboratories; Svoboda, Johnson, Neuss, and Gonman at the Lilly Research Laboratories) investigated the antidiabetic property of periwinkle extracts but did not find substantial hypoglycemic activity. The intense research on the extracts of periwinkle led to the isolation of an alkaloid, which reduced the WBCs and depleted bone marrow in rats. The new alkaloid was named as vincaleukoblastine because of its origin and effect on WBCs; subsequently named as vinblastine. Thorough phytochemical investigation of the extract over a period of a decade led to the discovery of anticancer alkaloids including vinblastine **(9)**, vincristine **(10)**, vinleunosine, and vinrosidine (Johnson et al., 1963; Noble, 1990, 2016). The vinca alkaloids are terpenoid indoles obtained from the coupling of catharanthine monomer and vindoline and are mainly used in chemotherapy regimens due to their ability to reduce the number of white blood cells in acute lymphoblastic leukemia and nephroblastoma. Vinca alkaloids represent the second most used class of anticancer drugs in the treatment of various malignancies (Moudi et al., 2013).

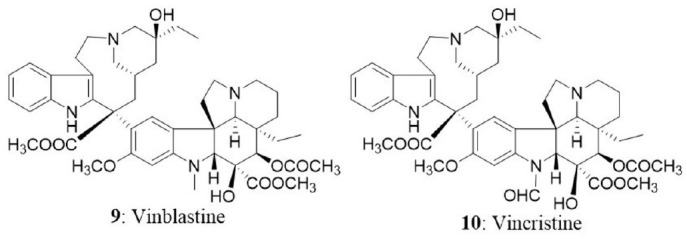



#### Mode of action

Vinca alkaloids target cell cycle progression by interfering with activities of the microtubule cytoskeleton. Microtubules are the cytoskeletal elements made up of tubulin heterodimers which are associated with transportation of cargo within the cell, motility of the cell itself, and progression of the cell cycle (Downing, 2000). In the structural organization of microtubules, α and β tubulins arrange themselves alternatively to form a protofilament and α subunits are projected toward minus end and β subunits toward plus end (confers polarity). Further, protofilaments arrange in a parallel fashion to form a hollow cylindrical structure called microtubule (Kirschner, 1980; Dumontet and Jordan, 2010). The microtubules grow and shrink rapidly by addition and removal of tubulin subunits by a phenomenon referred as dynamic instability of microtubules. The free subunits are added up at plus end with the subsequent release of subunits at minus end which results in the steady-state maintenance of microtubule length (Alberts, 2017). The shortening and lengthening of a microtubule are essential to pull sister chromosomes during mitosis and push out membranes respectively (Wilson et al., 1999). Impeding the dynamics and stability of microtubules can block cell division in multiple ways. Vinca alkaloids are found to interact with the vinca domain of β tubulin in order to destabilize the microtubule structure. The binding site is present toward the plus end of microtubule and vinblastine suppresses dynamic instability at plus ends (Wilson et al., 1999). Previous findings suggested that vinca derivatives inhibit cell cycle at metaphase of mitosis (Jordan et al., 1991). They bind to tubulin intracellularly, resulting in the subsequent dissolution of microtubules, and prevent cells from assembling the spindles needed for the division, thus arresting cells in mitosis, which is necessary to mediate their cytotoxic effects (Jordan et al., 1991; Newman and Cragg, 2007). Primarily vinca alkaloids interfere with the formation of microtubule and mitotic spindle dynamics coupled with the disruption of intracellular transport. Overall, vinca alkaloids impart their antineoplastic effect by serving as microtubule-destabilizing agents. Vincristine and vinblastine are approved for the treatment of Hodgkin lymphoma and structural analogs of vinca alkaloids (Vinflunine, Vinorelbine, Anhydrovinblastine) targeting tubulin polymerization are in phase-II/III trials for the treatment of breast cancer and carcinoma (Kaur et al., 2014).

#### Vinca alkaloids from endophytic fungi

An endophytic fungus, *Alternaria* sp., isolated from the phloem of *Catharanthus roseus* was reported to produce vinblastine (Guo et al., 1998). Later, the endophyte, *F. oxysporum* isolated from the phloem of *C. roseus* was found to produce vincristine (Zhang et al., 2000). An endophytic fungus from the leaves of *C. roseus* was also reported to produce vincristine (Yang et al., 2004). The endophytic fungus, *F. solani* from *C. roseus* was screened for vinca alkaloids using thin layer chromatography and electron spray ionization-mass spectroscopic analysis. The fungus was identified to produce vincristine and vinblastine (Kumar et al., 2013). The amount of vinblastine and vincristine in the culture filtrate was found to be 76 and 67 μg/L, respectively. The endophytic fungus *Talaromyces radicus* from *C. roseus* produced 670 μg/L of vincristine in modified M2 medium and 70 μg/L of vinblastine in PDB medium. Vincristine was partially purified and evaluated for cytotoxicity in HeLa, MCF7, A549, U251, and A431 cells. The treatment of vincristine resulted in the dose-dependent growth inhibition in HeLa, MCF7, A549, U251, and A431 with IC_50_ values of 4.2, 4.5, 5.5, 5.5, and 5.8 μg/mL, respectively. However, the normal cells (HEK293) were not significantly affected (Palem et al., 2015).

## Cytotoxic compounds from endophytic fungi associated with terrestrial plants

The secondary metabolites from endophytic fungi have been widely recognized as potent antitumor agents. The secondary metabolite and bioactive compounds from endophytic fungi are screened for their antiproliferative activity against different cancer cell lines (Please refer Tables 4–6 for examples). Preliminary screening of extracts or compounds using cytotoxicity assays allows the rapid identification of compounds with anticancer activity. It is also essential to identify compounds that discriminate between cancer and normal cells, and specifically induce cytotoxicity in cancer cells. Specific cytotoxicity is imperative in cancer therapy, as the standard chemotherapy often affects actively dividing normal cells. However, few anticancer agents may be carcinogenic (Blagosklonny, 2005). Hence, toxicity screening is one of the major steps employed to identify anticancer agents (Parasuraman, 2011). In the next section, compounds isolated from endophytic fungi associated with terrestrial plants and their anticancer potential have been discussed.

**Table 4:** List of cytotoxic compounds isolated from endophytic fungi of terrestrial habitats.

**Sl No**	**Host plant**	**Fungal endophyte**	**Isolated cytotoxic compound/s**	**Tested cell line/s**	**Cytotoxicity**	**References & Units of cytotoxicity**
1	*Tectona grandis*	*Phomopsis* sp.	Phomoxanthone A	KB BC-1 Vero	0.99 0.51 1.4	Isaka et al., 2001 IC_50_ (μg/mL)
			Phomoxanthone B	KB BC-1 Vero	4.1 0.7 1.8	
2	*Knema laurina*	*Acremonium* sp.	Brefeldin A	KB BC-1 NCI-H187	0.18 0.04 0.11	Chinworrungsee et al., 2008 IC_50_ (μM)
			8-Deoxy-trichothecin	KB BC-1 NCI-H187	>62.81 0.88 1.48	
			Trichothecolone	KB BC-1 NCI-H187	12.90 10.06 11.31	
			7α-Hydroxytrichodermol	KB BC-1 NCI-H187	>75.10 2.37 1.73	
			7α-Hydroxyscirpene	KB BC-1 NCI-H187	8.47 21.53 27.76	
3	*Polygonum senegalense*	*Alternaria* sp.	Alternariol Alternariol 5-O-sulfate Alternariol 5-O-methyl ether Altenusin Desmethylaltenusin	L5178Y	1.7 4.5 7.8 6.8 6.2	Aly et al., 2008 EC_50_ (μg/mL)
4	*Platycladus orientalis*	*Phyllosticta spinarum*	Tauranin	NCI-H460 MCF7 SF-268 PC-3M MIA Pa Ca-2	4.3 1.5 1.8 3.5 2.8	Wijeratne et al., 2008 IC_50_ (μM)
5	*Glochidion ferdinandi*	*Eupenicillium* sp.	Trichodermamide C	HCT116 A549	0.68 4.28	Davis et al., 2008 IC_50_ (μM)
6	*Hopea hainanensis*	*Penicillium* sp.	Monomethylsulochrin	KB HepG2	30.0 30.0	Wang et al., 2008 IC_50_ (μg/mL)
			Rhizoctonic acid	KB HepG2	20.0 25.0	
			Asperfumoid 3	KB HepG2	20.0 15.0	
			3,5-Dichloro-p-anisic acid 6	KB HepG2	5.0 10.0	
7	*Aquilaria sinensis*	*Preussia* sp.	Spiropreussione A	A2780 BEL-7404	2.4 3.0	Chen et al., 2009 IC_50_ (μM)
8	*Etlingera littoralis*	*Eutypella* sp.	Ent-4(15)-eudesmen-11-ol-1-one	NCI- H187 MCF7 KB Vero	11 20 32 32	Isaka et al., 2009 IC_50_ (μM)
9	*Mimosops elengi*	*Ascomycetes*	Ergoflavin	ACHN NCI-H460 Panc1 HCT116 Calu1	1.2 4.0 2.4 8.0 1.5	Deshmukh et al., 2009 IC_50_ (μM)
10	*Salvia officinalis*	*Chaetomium* sp.	Cochliodinol Isocochliodinol	L5178Y	7.0 71.5	Debbab et al., 2009b EC_50_ (μg/mL)
11	*Camellia sinensis*	*Pestalotiopsis fici*	Pestaloficiol L	HeLa MCF7	8.7 17.4	(Liu L. et al., 2009) IC_50_ (μM)
12	*Roystonea regia*	*Pestalotiopsis* *photiniae*	Photinides A Photinides B Photinides C Photinides D Photinides E Photinides F	MDA-MB-231	24.4 24.2 23.1 24.4 24.6	Ding et al., 2009 (% inhibitory rate at 10 μg/mL)
13	*Mentha pulegium*	*Stemphylium* *globuliferum*	6-O-Methylalteranin Alterporriol G/H	L5178Y	4.2 2.27	Debbab et al., 2009a EC_50_ (μg/mL)
14	*Ginkgo biloba*	*Chaetomium globosum*	Chaetomugilin A Chaetomugilin D Chaetoglobosin A Chaetoglobosin C	Brine shrimp	78.3 75.2 83.4 75.3	Qin et al., 2009 (% mortality rate at 10 μg/mL)
15	*Liculaspinosa*	*Xylaria* sp.	Eremophilanolide 1 Eremophilanolide 2 Eremophilanolide 3	KB MCF7 NCI-H187	3.8–21	Isaka et al., 2010 IC_50_ (μM)
16	*Musa acuminata*	*Phomopsis* sp.	Oblongolides Z	KB BC NCI-H187 Vero	37 26 32 60	Bunyapaiboonsri et al., 2010 IC_50_ (μM)
17	Tree from Gassan stock farm	*Allantophomopsis lycopodina*	Allantopyrone A Islandic acid-II methyl ester	HL60	0.32 6.55	Shiono et al., 2010 IC_50_ (μM)
18	*Artemisia annua*	*Chaetomium globosum*	Chaetoglobosins V	KB K562 MCF7 HepG2	23.53 >30 27.86 >30	Zhang J. et al., 2010 IC_50_ (μg/mL)
			Chaetoglobosins W	KB K562 MCF7 HepG2	21.17 >30 >30 27.87	
19	*Rehmannia glutinosa*	*Massrison* sp.	Massarigenin D	LO2 HepG2 MCF7 A549	19.6 20.8 11.2 14.4	Sun et al., 2011 IC_50_ (μg/mL)
			Spiromassaritone	LO2 HepG2 MCF7 A549	7.2 5.6 6.8 9.8	
			Paecilospirone	LO2 HepG2 MCF7 A549	12.4 10.4 7.6 6.8	
20	*Cinnamomum kanehirae*	*Fusarium oxysporum*	Beauvericin	PC-3 Panc-1 A549	49.5 47.2 10.4	Wang et al., 2011 IC_50_ (μM)
21	*Dysoxylumbi nectariferum* Hook.f	*Fusarium proliferatum*	Rohitukine	HCT-116 MCF7	10 10	Mohana Kumara et al., 2012 IC_50_ (μg/mL)
22	*Cinnamomum* sp.	*Annulohypoxylon* *squamulosum*	Annulosquamulin	MCF7 NCI-H460 SF-268	3.19 3.38 2.46	Cheng et al., 2012 IC_50_ (μg/mL)
			(3S)-7-hydroxymellein	MCF7 NCI-H460 SF-268	2.78 3.17 2.38	
23	*Erythrophleum fordii* Oliver	*Alternaria tenuissima*	Tenuissimasatin	HCT-8	>1	Fang et al., 2012 IC_50_ (μmol/L)
			(6aR, 6bS, 7S)-3, 6a, 7, 10-tetrahydroxy-4, 9-dioxo-4, 6a, 6b, 7, 8, 9-hexahydroperylene	HCT-8	1.78	
24	*Curcuma wenyujin*	*Chaetomium globosum* Kunze	Chaetoglobosin X	H22 MFC	3.125 6.25	Wang et al., 2012 IC_50_ (μg/mL)
25	*Erythrina variegata*	*Alternaria* sp.	Altersolanol Macrosporin 1,2,4,5-tetrahydroxy-7-methoxy-2-methyl-1,2,3,4-tetrahydroanthracene-9,10-dione	Angiogenesis assays	–	Pompeng et al., 2013
26	*Tamarix chinensis*	*Penicillium* sp. SXH-65	Arisugacin B Arisugacin F	HeLa HL-60 K562	24–60	Sun et al., 2014 IC_50_ (μM)
27	*Taxus chinensis* var. *mairei*	*Perenniporia* *tephropora* Z41	Perenniporin A	HeLa SMMC-7721Panc-1	30.44 45.49 44.22	Wu et al., 2013 IC_50_ (μg/mL)
28	*Capsicum annum*	*Alternaria alternata*	Alternariol-10-methyl ether	HL-60 A549 PC-3	85 >100 >100	Devari et al., 2014 IC_50_ (μM)
				HeLa A431 MIA PaCa-2 T47D	>100 95 >100 >100	
29	*Ipomoea batatas*	*Aspergillus glaucus*	2, 14-dihydrox-7-drimen-12, 11-olide	HepG2 MCF7	61 41.7	Mohamed et al., 2013 IC_50_ (μg/mL)
30	*Gloriosa superba*	*Aspergillus* sp.	Colchatetralene	THP-1 MCF7	30 50	Budhiraja et al., 2013 IC_50_ (μg/mL)
31	*Panax ginseng*	*Penicillium melinii*	Ginsenocin	MKN45 LOVO A549 MDA-MB-435 HepG2 HL-60	1.91 2.20 5.03 1.39 2.34 0.49	Zheng et al., 2013 IC_50_ (μg/mL)
			Penicillic acid	MKN45 LOVO A549 MDA-MB-435 HepG2 HL-60	2.85 2.21 7.46 6.28 3.67 0.80	
		*Penicillium janthinellum*	Brefeldin A	MKN45 LOVO A549 MDA-MB-435 HepG2 HL-60	< 0.001 0.12 0.04 < 0.001 < 0.001 < 0.001	
32	*Clidemia hirta*	*Cryptosporiopsis* sp.	(R)-5-hydroxy-2-methylchroman-4-one	HL-60	4	Zilla et al., 2013 IC_50_ (μg/mL)
33	*Nerium oleander* L.	*Cochliobolus kusanoi*	Oosporein	A549	21	Alurappa et al., 2014 IC_50_ (μM)
34	*Tripterygium wilfordii*	*Penicillium* sp. HSZ-43	Penifupyrone Funicone Deoxyfunicone 3-O- Methylfunicone	KB	4.7 13.2 22.6 35.3	Chen et al., 2014 IC_50_ (μM)
35	*Salicornia bigelovii* Torr	*Fusarium equiseti*	Diglucotol	MCF7 MDA-MB-231Caco-2	97.56 92.35 99.39	Wang et al., 2014 EC_50_ (μM)
			Cerevisterol	MCF7 MDA-MB-231Caco-2	32.4 41.5 37.56	
			Ergosterol peroxide	MCF7 MDA-MB-231Caco-2	64.5 52.4 77.56	
36	*Corylus avellana*	*Phomopsis amygdale*	Pestalotin	HT-29 PC-3 MDA-MB-231 HEK293	16.69 20.54 41.7 11.83	Akay et al., 2014 IC_50_ (μg/mL)
			(S)-4-butoxy-6-((S)-1-hydroxypentyl)-5,6-dihydro-2H-pyran-2-one	HT-29 PC-3 MDA-MB-231 HEK293	82.18 132.23 24.26 13.84	
37	*Ajuga decumbens* Thunb	*Botryotinia* *fuckeliana* A-S-3	Phenochalasin B	SMMC-7721 A549 HepG2 MCF7	7.43 0.83 2.2 0.1	Lin et al., 2015 IC_50_ (μM)
			[12]-cytochalasin	SMMC-7721 A549 HepG2 MCF7	0.88 0.66 0.62 0.59	
38	*Moringa oleifera*	*Cladosporium oxysporum*	Taxol	HCT15	3.5	Gokul Raj et al., 2015 IC_50_ (μM)
39	*Xanthium sibiricum*	*Eupenicillium* sp. LG41	Eupenicinicol C Eupenicinicol D	THP-1	– 8	Li and Kusari, 2017 IC_50_ (μM)
40	*Pinellia ternata*	*Penicillium brefeldianum*	6,7- dehydropaxilline	MDA-MB-231 U2-OS HepG2	>50 >50 >50	Gao et al., 2017 IC_50_ (μmol/L)
			Spirotryprostatin F	MDA-MB-231 U2-OS HepG2	35.5 >50 14.1	
			N-demethyl melearoride A	MDA-MB-231 U2-OS HepG2	>50 >50 36.6	

*Among the isolated compounds, only potent compounds are included in the column “isolated compound/s”*.

*Cell lines: OVCAR-3 (Ovarian cancer), Jurkat (Human T cell leukemia), Jurkat-JR16 (Jurkat cells overexpressing Bcl2), LOVO (Colon cancer), A549 (Lung cancer), MDA-MB-435 (Breast cancer), HepG2 (Liver cancer), HL-60 (Promyelocytic leukemia), KB (Oral cancer), KBv200 (Multidrug resistant cells), MCF7 (Breast cancer), Caco-2 (Colon cancer), PC-3 (Prostate cancer), HeLa (Cervical cancer), A431 (Skin cancer), MDA-MB-231 (Breast cancer), MIA PaCa-2 (Pancreatic cancer), T47D (Breast cancer), HT 29 (Colon cancer), HEK293 (Kidney), SMMC-7721 (Liver cancer), HCT 15 (Colon cancer), THP-1 (Acute monocytic leukemia), U2-OS (Bone cancer), L5178Y (Mouse lymphoma), HEp2 (Cervical cancer), PC-12 (Rat adrenal gland cancer), KV/MDR (Multi-drug resistant oral cancer), SW620 (Colon cancer), 95-D (Lung cancer), Kato-3 (Gastric cancer), CaSki (Cervical cancer), NCI-H460 (Lung cancer), SW1990 (Pancreatic cancer), DU145 (Prostate cancer), BC-1 (Lymphoma), Vero (African green monkey kidney fibroblasts), NCI-H187 (Lung cancer), SF-268 (Brain cancer), HCT 116 (Colon cancer), A2780 (Ovarian cancer), BEL-7404 (Liver cancer), ACHN (Kidney cancer), Panc-1 (Pancreatic cancer), Calu1 (Lung cancer), K562 (Chronic myelogenous leukemia), LO2 (Liver), HCT-8 (Colon cancer), H22 (Mouse liver cancer), U-87 (Brain cancer), BGC-823 (Gastric cancer), U251 (Brain cancer), A-2 (Lung cancer), PC-3M (Metastatic prostate cancer), MDCK (Dog kidney), OVCAR-5 (Ovarian cancer), CV1 (Kidney), MFC (Mouse gastric cancer), SK-OV-3 (Ovarian cancer), SK-MEL-2 (Skin cancer), XF-498 (Brain cancer), P388 (Mouse lymphoma), MKN45 (Gastric cancer), and U937 (Histiocytic lymphoma)*.

**Table 5:** List of anticancer compounds isolated from endophytic fungi from mangrove habitats.

**Sl No**	**Host plant**	**Fungal endophyte**	**Isolated cytotoxic compound/s**	**Tested cell line/s**	**Cytotoxicity**	**References & Units of cytotoxicity**
1	*Excoecaria agallocha*	*Phomopsis* sp. ZSU-H76	2-(7′-hydroxyoxooctyl)-3-hydroxy-5-methoxybenzeneacetic acid ethyl ester	HEp2 HepG2	25 30	Huang et al., 2009 IC_50_ (μg/mL)
2	*Rhizophora mucronata*	*Pestalotiopsis* sp.	Cytosporones J-N Pestalasins A-E Pestalotiopsoid A	L5178Y HeLa PC12	Not Active up to 10 μg/mL	Xu et al., 2009a
3	*Rhizophora mucronata*	*Pestalotiopsis* sp.	Pestalotiopsone A Pestalotiopsone B Pestalotiopsone C Pestalotiopsone D Pestalotiopsone E Pestalotiopsone F	L5178Y	NA NA NA NA NA 26.89	Xu et al., 2009a IC_50_ (μM)
4	*Not mentioned*	*Mangrove endophytic fungus No. ZSU44*	Secalonic acid D	HL60 K562	0.38 0.43	Zhang J. Y. et al., 2009 IC_50_ (μM)
5	*Excoecaria agallocha*	*Phomopsis* sp.	Phomopsis-H76 A Phomopsis-H76 B Phomopsis-H76 C	KB KBv200 MCF7	All the compounds are inactive against all the tested cell lines	Yang et al., 2010
6	Kandelia woody tissue	*Halorosellinia* sp. *Guignardia* sp.	1-hydroxy-3-methyl anthracene-9,10-dione	KB KBv200	3.17 3.21	Zhang J. Y. et al., 2010 IC_50_ (μM)
7	*Sonneratia apetala*	Zh6-B1 (unidentified)	3R,5R-Sonnerlactone 3R,5S-Sonnerlactone	KV/MDR	42.4 41.6	Li et al., 2010 % inhibition at 100 μM
8	*Xylocarpus granatum*	XG8D (unidentified)	Merulin A	BT474 SW620	4.98 4.84	Chokpaiboon et al., 2010 IC_50_ (μg/mL)
			Merulin B	BT474 SW620	>10 >10	
			Merulin C	BT474 SW620	1.57 4.11	
9	*Acanthus ilicifolius*	*Penicillium* sp.	Penicinoline	95-D HepG2 HeLa KB KBv200 HEp2	0.57 6.5 >100 >100 >100 >100	Shao et al., 2010 IC_50_ (μg/mL)
10	Unidentified mangrove (Taiwan Strait)	*Paecilomyces* sp.	Paeciloxocins A Paeciloxocins B	HepG2	1 65	Wen et al., 2010 IC_50_ (μg/mL)
11	*Excoecaria agallocha*	*Penicillium expansum*	Expansols A	A549 HL-60	NR 15.7	Lu et al., 2010 IC_50_ (μM)
			Expansols B	A549 HL-60	1.9 5.4	
12	*Kandelia candel*	*Fusarium* sp.	5-O-methyl-2′-methoxy-3′-methylalpinumisoflavone	HEp2 HepG2	4 11	Huang et al., 2010 (μmol/mL)
13	*Aegiceras corniculatum*	*Alternaria* sp. ZJ9-6B	Alterporriol K	MDA-MB-435 MCF-7	26.97 29.11	Huang et al., 2011 IC_50_ (μM)
			Alterporriol L	MDA-MB-435 MCF7	13.11 20.04	
			Alterporriol M	MDA-MB-435 MCF-7	NT NT	
14	*Rhizophora mucronata*	*Irpex hynoides*	Ethyl acetate extract was used for cytotoxic studies. Tetradecane was isolated from the extract.	HEp2	125	Bhimba et al., 2011 IC_50_ (μg/mL)
15	*Rhizophora annamalayan*	*Fusarium oxysporum*	Taxol	NT	NT	Elavarasi et al., 2012
16	*Bruguiera gymnorrhiza*	*Rhytidhysteron rufulum*	Rhytidchromones A	MCF7 HepG2 Kato-3 CaSki	19.3 >25 23.3 >25	Chokpaiboon et al., 2016 IC_50_ (μM)
			Rhytidchromones B	MCF7 HepG2 Kato-3 CaSki	>25 >25 21.4 >25	
			Rhytidchromones C	MCF7 HepG2 Kato-3 CaSki	>25 >25 >25 >25	
			Rhytidchromones D	MCF7 HepG2 Kato-3 CaSki	>25 >25 16.8 >25	
			Rhytidchromones E	MCF7 HepG2 Kato-3 CaSki	17.7 >25 16 >25	

*Among the isolated compounds, only potent compounds are included in the column “isolated compound/s”*.

*NA, Not active; NR, Not reported; NT, Not tested*.

**Table 6:** List of marine cytotoxic endophytic fungal metabolites.

**Sl No**	**Host plant**	**Fungal endophyte**	**Isolated cytotoxic compound/s**	**Tested cell line/s**	**Cytotoxicity**	**References & Units of cytotoxicity**
1	Seaweed	*Talaromyces* sp	Kasanosin A Kasanosin B	NT	–	Kimura et al., 2008
2	*Acanthophora spicifera*	*Curvularia* sp.	Apralactone A (+)-(10E,15R)-10,11-dehydrocurvularin (+)-(15R)-12-hydroxy-10,11-E-dehydrocurvularin (+)-(15R)-13-hydroxy-10,11-E-dehydrocurvularin (+)-(11S,15R)-11-hydroxycurvularin (+)-(11R,15R)-11-hydroxycurvularin (+)-(15R)-12-oxocurvularin Compound 8	36 cancer cell lines	0.28–>32.64	Greve et al., 2008 IC_50_ (μM)
3	*Ectyplasia perox*	*Phoma* sp.	Epoxyphomalin A Epoxyphomalin B	36 Human cancer cell lines	0.017–11.42	Mohamed et al., 2009 IC_50_ (μg/mL)
4	*Petrosia* sp	*Aspergillus Versicolor*	Sterigmatocystin	A549 SK-OV-3 SK-MEL-2 XF-498 HCT-15	1.86 2.53 1.22 2.75 4.61	Lee et al., 2010 IC_50_ (μg/mL)
			Averantin	A549 SK-OV-3 SK-MEL-2 XF-498 HCT-15	3.15 3.88 3.57 3.04 3.13	
			Methylaverantin	A549 SK-OV-3 SK-MEL-2 XF-498 HCT-15	0.64 1.17 1.10 0.41 0.49	
			Nidurufin	A549 SK-OV-3 SK-MEL-2 XF-498 HCT-15	1.83 3.39 3.16 1.78 2.20	
5	*Sargassum kjellmanianum*	*Aspergillus ochraceus*	7-nor-ergosterolide	NCI-H460 SMMC-7721 SW1990	5 7 28	Cui et al., 2010a IC_50_ (μg/mL)
			3β,11α-dihydroxyergosta-8,24(28)-dien-7-one	NCI-H460 SMMC-7721 SW1990	NR 28 NR	
6	*Ulva pertusa*	*Chaetomium globosum*	Cytoglobosins A Cytoglobosins B Cytoglobosins C Cytoglobosins D Cytoglobosins E Cytoglobosins G	A549	>10 >10 2.26 2.25 >10 >10	Cui et al., 2010b IC_50_ (μM)
7	*Limonium tubiflorum*	*Penicillium* sp.	11β-methoxycurvularin	K562 Jurkat U937	12.4 3.9 1.8	Aly et al., 2011
			11a-methoxycurvularin 4	K562 Jurkat U937	41.1 2.3 4.6	
			Dehydrocurvularin 6	K562 Jurkat U937	37.0 5.5 2.5	
			1-chloro-2,4-dihydroxy-5-methoxy-7-methylanthraquinone	K562 Jurkat U937	24.8 13.3 7.6	

*Among the isolated compounds, only potent compounds are included in the column “isolated compound/s”*.

*NA, Not active; NR, Not reported; NT, Not tested*.

Two new xanthone dimers, phomoxanthone A **(11)** and B **(12)** were isolated from the endophytic fungus *Phomopsis* sp., BCC 1323 from *Tectona grandis* at Mee Rim district, Chiang Mai Province, Northern Thailand. The compounds exhibited remarkable cytotoxicity towards KB, BC-1 cells, and non-malignant Vero cells. Phomoxanthone A was relatively more potent than phomoxanthone B with IC_50_ values of 0.99, 0.51, and 1.4 μg/mL against KB, BC-1, and Vero cells, respectively. Moreover, phomoxanthone A and B displayed selectively potent cytotoxicity in BC-1 cells with IC_50_ values 0.51 and 0.70 μg/mL, respectively (Isaka et al., 2001).

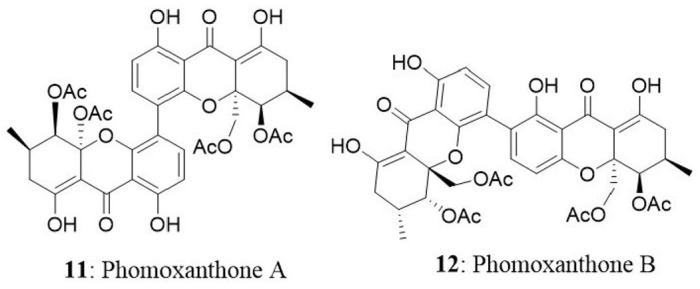



A new species of *Acremonium* was isolated from the twig of *Knema laurina* (Thai medicinal plant) in the forest areas of Nakhon Ratchasima Province, Thailand. The endophytic fungus was found to produce brefeldin A **(13)** 8-deoxy-trichothecin, trichothecolone, 7-hydroxytrichodermol, and 7-hydroxyscirpene. Brefeldin A exhibited potent cytotoxic activity against KB, BC-1, and NCI-H187 with IC_50_ values of 0.18, 0.04, and 0.11 μM, respectively (Chinworrungsee et al., 2008). Altersolanol **(14)**, a hydroxylated tetrahydro anthraquinone, macrosporin **(15)**, and 1,2,4,5-tetrahydroxy-7-methoxy-2-methyl-1,2,3,4-tetrahydroanthracene-9,10-dione **(16)** were isolated from an *Alternaria* endophytic fungus. The fungus is collected from a healthy leaf of *Erythrina variegata* in Samutsakorn Province, Thailand. Altersolanol showed potent antiangiogenic activity by inhibiting endothelial cell proliferation, tube formation, and migration. Altersolanol also suppressed the formation of blood vessels in *ex vivo* and *in vivo* models. Taken together, altersolanol was found to be a good antiangiogenic agent and a promising candidate in development of therapeutics against cancer and other pro-angiogenesis-related diseases (Pompeng et al., 2013).

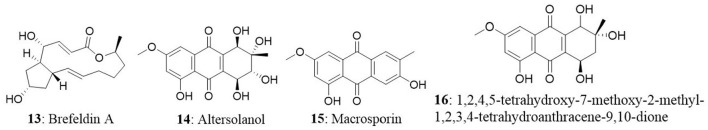



The endophytic fungus *Preussia* sp., was obtained from a mature stem of *Aquilaria sinensis* (Thymelaeaceae) collected from Guangxi Medicinal Arboretum. The endophyte produced three spirobisnaphthalenes (spiropreussione A, spiropreussione B, and spiropreussomerin A) possessing two naphthalene-derived C-10 units' bridges through a spiroketal linkage. Among the novel spirobisnaphthalenes, spiropreussione A **(17)** exhibited *in vitro* cytotoxic activity against the A2780 and the BEL-7404 cell lines with IC_50_ values of 2.4 and 3.0 μM, respectively. However, spiropreussione A was less active against the HCT-8, BGC-823, and A549 cell lines with IC_50_ values more than 10 μM (Chen et al., 2009). The endophytic fungus *Eutypella* sp. from *Etlingera littoralis* produced two new γ-lactones (eutypellins A and B), two ent-eudesmane sesquiterpenes (ent-4(15)-eudesmen11-ol-1-one and ent-4(15)-eudesmen-1R,11-diol), and three known pimarane diterpenes (diaporthein B, scopararane A, and libertellenone C). Among the isolated compounds, ent-4(15)-eudesmen-11-ol-1-one **(18)** showed weak cytotoxicity against NCI-H187, MCF7, KB, and Vero cell lines with IC_50_ values of 11, 20, 32, and 32 μM, respectively (Isaka et al., 2009). In another study, seven novel eremophilane-type sesquiterpenes and the known mairetolide F were isolated from the endophyte *Xylaria* sp. The fungus was isolated from *Licula spinose* at Trang Province, Thailand. The compounds eremophilanolide 1, 2 and 3 **(19-21)** exhibited moderate cytotoxicity with IC_50_ values ranging between 3.8 and 21 μM against the KB, MCF7, and NCI-H187 cell lines (Isaka et al., 2010).

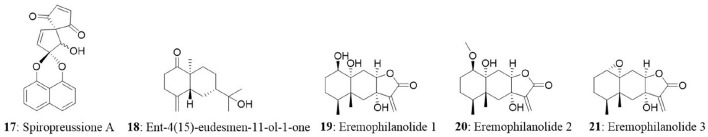



The endophyte *Phomopsis* sp. was isolated from the leaf of *Musa acuminata* collected at Doi Suthep Pui National Park, Chiang Mai Province, Thailand. The fungus produced six new oblongolides and seven known compounds. Among the new compounds, oblongolides Y **(22)** and Z **(23)** were cytotoxic against the tested cells. The compound 22 was superior over its counterpart with the IC_50_ values of 37, 26, 32, and 60 μM against KB, BC and NCI-H187, and Vero cell lines, respectively. The positive control, doxorubicin had the IC_50_ values of 0.24 μM for KB, 0.30 μM for BC, and 0.08 μM for NCI-H187 (Bunyapaiboonsri et al., 2010). In the next study, the fungus was found to produce four new compounds named oblongolides C1, P1, X1, and 6-hydroxyphomodiol together with eight known compounds including oblongolides B, C, D, O, P, U, (3R,4aR,5S,6R)-6-hydroxy-5-methylramulosin, and (3R)-5-methylmellein. The new compounds exhibited weak selective cytotoxicity against HepG2 cells with an inhibitory rate ranging between 16.89 and 28.59% (Lin et al., 2011). An endophytic *Alternaria* sp., associated with the Egyptian medicinal plant *Polygonum senegalense* was isolated. When the endophyte was cultured on the solid rice medium, four new compounds (3′-hydroxyalternariol 5-O-methyl ether, desmethylaltenusin, alterlactone, and alternaric acid) and five known compounds [alternariol **(24)**, alternariol 5-O-methyl ether, altenusin, talaroflavone, and altenuene) were obtained. And when the fungus was grown on liquid Wickerham medium, two new compounds (alternariol 5-O-sulfate **(25)** and alternariol 5-O-methyl ether **(26)**] and six known compounds (alternariol, alternariol 5-O-methyl ether, altenusin, 2,5-dimethyl-7-hydroxychromone, tenuazonic acid, and altertoxin I) were isolated. Overall, fungal extract analysis revealed the presence of 15 natural compounds, out of which seven were new compounds. Compounds 24, 25, and 26 showed cytotoxicity against L5178Y cells with EC_50_ values ranging between 1.7 and 7.8 μg/mL, compared with the positive control (kahalalide F), which had the EC_50_ value of 6.3 μg/mL. The two bicyclic acid derivatives, Alutenusin **(27)** and desmethylaltenusin **(28)** exhibited moderate cytotoxic activity against L5178Y cells, with EC_50_ values of 6.8 and 6.2 μg/mL, respectively (Aly et al., 2008).

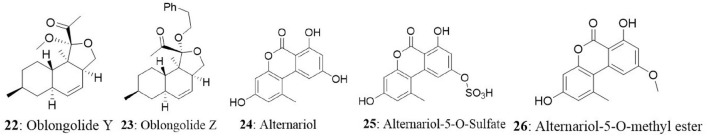



Ergoflavin **(29)** belongs to the class of compounds called ergochromes, and it was first isolated from the ergot fungus (*Claviceps purpurea*) followed by identification in *Phoma terrestris, Pyrenochaeta terrestris, Penicillium oxalicum*, and *Aspergillus* sp. The ergoflavin was isolated from endophyte collected from the leaf of *Mimusops elengi*. The isolated ergoflavin inhibited human TNF-α and IL-6 with IC_50_ values of 1.9 and 1.2 μM, respectively. Further investigation revealed that ergoflavin induces cytotoxicity in ACHN, NCI-H460, Panc-1, HCT116, and Calu1 cell lines with IC_50_ values of 1.2, 4.0, 2.4, 8.0, and 1.5 μM, respectively (Deshmukh et al., 2009).

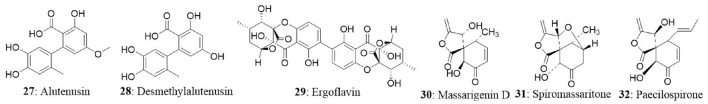



The endophytic fungus *Massrison* sp. was isolated from the roots of wild *Rehmannia glutinosa* collected from Wushe County, Henan Province, China. Extract from the culture broth revealed the presence of massarigenin D **(30)**, spiromassaritone **(31)**, and paecilospirone **(32)**. These compounds contain a rare spiro-5,6-lactone ring skeleton. The cytotoxic effects of these three compounds were tested against LO2, HepG2, MCF7, and A-2 cells. The compounds showed good cytotoxicity against the tested cells. Spiromassaritone displayed potent cytotoxicity against all the tested cells with an IC_50_ value ranging between 5.6 and 9.8 μg/mL (Sun et al., 2011).

The endophytic fungus was collected from stems of *Salvia officinalis* from the mountain of Beni-Mellal, Morocco. Chemical analysis of secondary metabolites revealed the presence of two known compounds namely, cochliodinol **(33)** and isocochliodinol **(34)**. The bioactive compounds were tested for cytotoxicity against L5178Y cells. Cochliodinol was potent than isocochliodinol, with an EC_50_ of 7.0 μg/mL compared with 71.5 μg/mL for isocochliodinol. The authors interpreted that, the difference in cytotoxicity of structurally related compounds was due to the position of prenyl substituents at the indole rings (Debbab et al., 2009b).

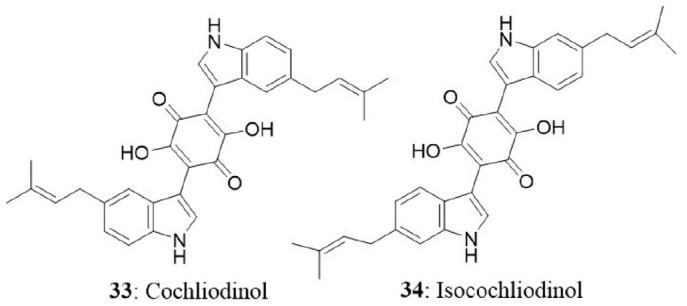



The fungal endophyte *Pestalotiopsis fici* was isolated from branches of *Camellia sinensis* in the suburb of Hangzhou, Zhejiang province, China. Seven new isoprenylated chromone derivatives namely pestaloficiols F-L were obtained. Among the newly isolated compounds, pestaloficiol J **(35)**, K **(36)**, L **(37)** exhibited cytotoxicity with IC_50_ values ranging between 8.7 and 99.3 μM for HeLa cell line. The IC_50_ values of other compounds ranged between 8.7 μM and >136.1 μM for HeLa cells and 17.4 μM and >153.8 μM for MCF7 cells. 5-fluorouracil was used as a positive control with IC_50_ values of 10 and 15 μM against HeLa and MCF7 respectively (Liu L. et al., 2009).

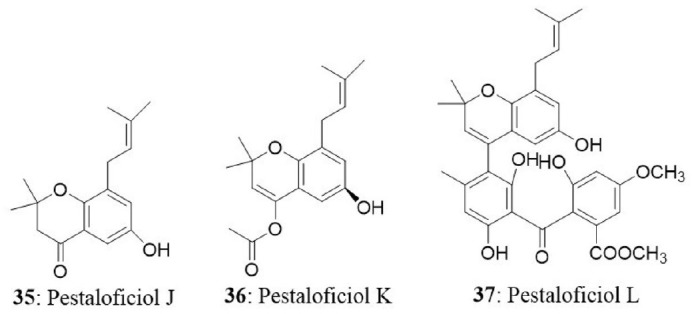



Six novel benzofuranone-derived γ-lactones called photinides A–F **(38-43)**, were isolated from the endophyte *Pestalotiopsis photiniae*. The endophyte was isolated from *Roystonea regia* collected from Jianfeng mountain, Hainan province, China. Photinides A-F exhibited modest and selective cytotoxicity against MDA-MB-231 cells with an inhibitory rate of 24.4, 24.2, 23.1, 24.4, and 24.6%, respectively, at a concentration of 10 μg/mL. No cytotoxicity was observed at 10 μg/mL against HeLa cells (Ding et al., 2009).

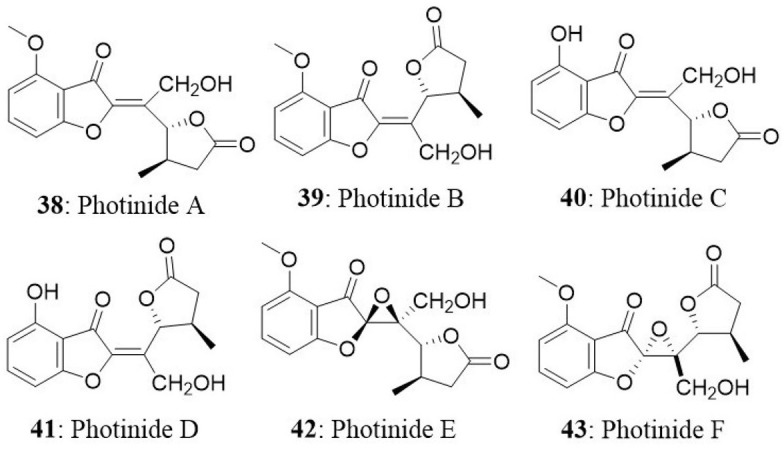



The endophyte *Stemphylium globuliferum* was isolated from the stem tissues of Egyptian medicinal plant *Mentha pulegium* collected in Morocco. Chemical investigation of extract revealed the presence of five novel compounds (alterporriol G, atropisomer alterporriol H, altersolanol K, altersolanol L, and stemphypyrone) and eight known compounds (6-O-methylalaternin, macrosporin, altersolanol A, alterporriol E, alterporriol D, alterporriol A, alterporriol B, and altersolanol J). The isolated compounds were tested for cytotoxic activity against L5178Y cells. An unresolved mixture of alterporriol G **(44)** and its atropisomer alterporriol H **(45)** exhibited the most potent cytotoxicity with an EC_50_ value of 2.7 μg/mL. The known compound 6-O-methylalaternin **(46)** also exhibited good cytotoxicity with an EC_50_ value of 4.2 μg/mL. The positive control (kahalalide F) exhibited an EC_50_ value of 6.3 μg/mL (Debbab et al., 2009a).

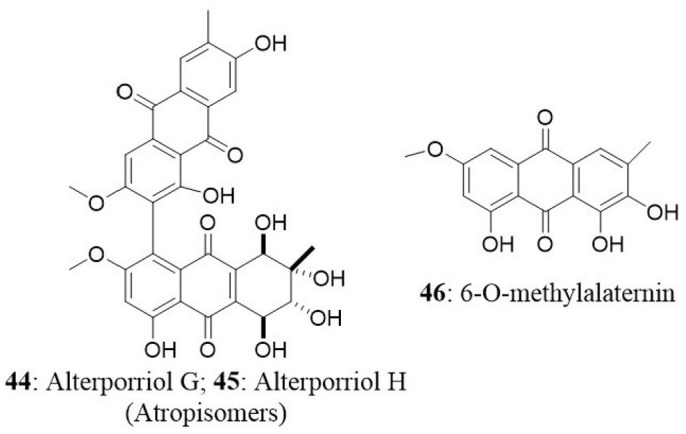



The endophyte *Phyllosticta spinarum* isolated from *Platycladus orientalis* from the Sonoran desert produced five new compounds [(+)-(5S,10S)-4′-hydroxymethylcyclozonarone, 3-ketotauranin, 3R-hydroxytauranin, 12-hydroxytauranin, and phyllospinarone] along with a known compound tauranin **(47)**. Tauranin exhibited cytotoxicity against NCI-H460, MCF7, SF-268, PC-3M, and MIA PaCa-2 cells with IC_50_ values of 4.3, 1.5, 1.8, 3.5, and 2.8 μM, respectively (Wijeratne et al., 2008). A newly modified dipeptide trichodermamide C **(48)** was isolated from the endophytic fungus *Eupenicillium* sp., from the rainforest tree *Glochidion ferdinandi* (Euphorbiaceae) in Australia. Trichodermamide C displayed cytotoxicity toward HCT116 and A549 cell lines with IC_50_ values of 0.68 and 4.28 μg/mL, respectively (Davis et al., 2008).

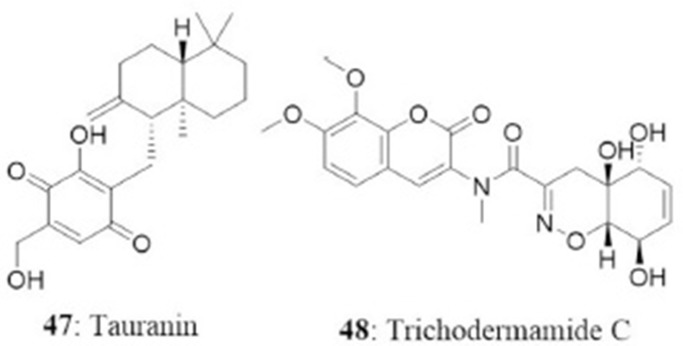



In another study, a new dihydrobenzofuran-2,4-dione derivative named as annulosquamulin **(49)** along with 10 known compounds were obtained from the endophytic fungus *Annulohypoxylon squamulosum*. The endophyte was isolated from the stem bark of the medicinal plant *Cinnamomum* sp. at Fu-Shan Botanical Garden, I-lan County, Taiwan. Annulosquamulin was evaluated for its cytotoxicity against MCF7, NCI-H460, and SF-268 cell lines. The IC_50_ values of annulosquamulin were 3.19, 3.38, and 2.46 μg/mL, respectively (Cheng et al., 2012).

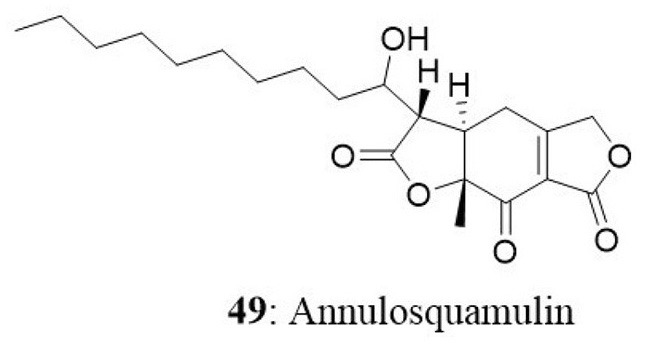



The endophytic fungal strain *Allantophomopsis lycopodina* was isolated from a tree branch on the Gassan stock farm, Yamagata, Japan, yielding a new natural product, allantopyrone A **(50)**, and a previously reported islandic acid-II methyl ester **(51)**. Both compounds exhibited good cytotoxic activity against HL60 cells with IC_50_ values of 0.32 and 6.55 μM, respectively. Cleavage of the genomic DNA is a hallmark event in the cells committed to apoptosis (Sebastian et al., 2016) which leads to the formation of cells with lesser DNA content called hypodiploid cells (Mohan et al., 2014, 2016; Roopashree et al., 2015). Treatment of HL60 cells with these compounds resulted in fragmentation of genomic DNA and N-acetyl-L-cysteine prevented this effect. Allantopyrone A demonstrated stronger cytotoxicity and apoptosis-inducing activity than the islandic acid-II methyl ester (Shiono et al., 2010).

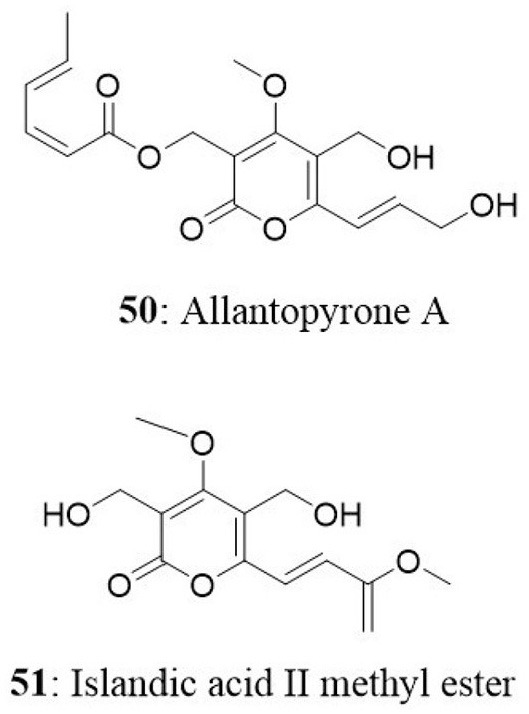



An endophytic fungus, *Cochliobolus kusanoi*, isolated from the stem of *Nerium oleander* L. produced the known compound oosporein **(52)**. The cytotoxicity of oosporein was evaluated toward A549 and presented the IC_50_ value of 21 μM (Alurappa et al., 2014). The cytotoxic potential of oosporein in the MDCK cell line and its role in oxidative stress *in vivo* and *in vitro* have been previously reported by our research group (Ramesha et al., 2015).

The endophytic fungus *Penicillium* sp., HSZ-43 was isolated from the leaves of Chinese traditional medicinal plant *Tripterygium wilfordii*. The endophyte was found to produce a novel compound, penifupyrone **(53)**, along with three known analogs: funicone **(54)**, deoxyfunicone **(55)**, and 3-O-methylfunicone **(56)**. The isolated compounds were tested for cytotoxicity against KB cells. Penifupyrone exhibited moderate cytotoxicity with an IC_50_ value of 4.7 μM. The IC_50_ values of funicone, deoxyfunicone and 3-O-methylfunicone were 13.2, 22.6, and 35.3 μM, respectively (Chen et al., 2014).

Meroterpenoids namely arisugacin I **(57)** arisugacin J **(58)**, arisugacin F **(59)**, arisugacin G **(60)**, arisugacin B **(61)**, territrem C **(62)**, and territrem B **(63)** were obtained from the endophytic fungus *Penicillium* sp. SXH-65. The fungus was isolated from the saline-alkaline soil along the coast of Laizhou Bay in Dongying, China. The isolated compounds were evaluated against a panel of cancer cell lines. Arisugacin B and Arisugacin F exhibited weak cytotoxicity against HeLa, HL-60, and K562 cell lines with IC_50_ values ranging from 24 to 60 μM (Sun et al., 2014). A new sesquiterpenoid, Perenniporin A **(64)**, from the endophytic fungus *Perenniporia tephropora* Z41 from *T. chinensis* var. *mairei* exhibited moderate cytotoxic activity with IC_50_ values ranging from 6 to 58 μg/mL against three human cancer cell lines (HeLa, SMMC-7721, and Panc-1). On the other hand, ergosterol **(65)** from the same source showed significant cytotoxicity against the HeLa, SMMC-7721, and Panc-1 cell lines with IC_50_ values of 1.16, 11.63, and 11.80 μg/mL (Wu et al., 2013).

A novel diglucotol **(66)**, together with five known compounds, cerebroside C **(67)**, N-acetyltryptamine **(68)**, 3β,5α,9α-trihydroxy-(22E,24R)-ergosta-7,22-dien-6-one **(69)**, cerevisterol **(70)**, and ergosterol peroxide **(71)**, were isolated from *Fusarium equiseti* from the salt-tolerant plant *Salicornia bigelovii* Torr. Diglucotol exhibited weak antiproliferative activity toward MCF7, MDA-MB-231, and Caco-2 cells with EC_50_ values of 97.56, 92.35, and 99.39 μM, respectively. Cerevisterol showed potent growth inhibitory activities against MCF7, MDA-MB-231, and Caco-2 cancer cells with EC_50_ values of 32.4, 41.5, and 37.56 μM, respectively. Ergosterol peroxide exhibited relatively less potency compared with cerevisterol and the EC_50_ values ranged between 52.4 and 77.56 μM (Wang et al., 2014). In another study, 2,14-dihydrox-7-drimen-12,11-olide **(72)** was isolated from *Aspergillus glaucus* from the leaves of *Ipomoea batatas*. Anticancer property of the isolated compound was evaluated in HepG2 and MCF7 cells. The isolated compound displayed moderate cytotoxic effect against HepG2 cells with an IC_50_ value of 61 μg/mL and possessed good cytotoxicity against MCF7 cells with IC_50_ values of 41.7 μg/mL (Mohamed et al., 2013).

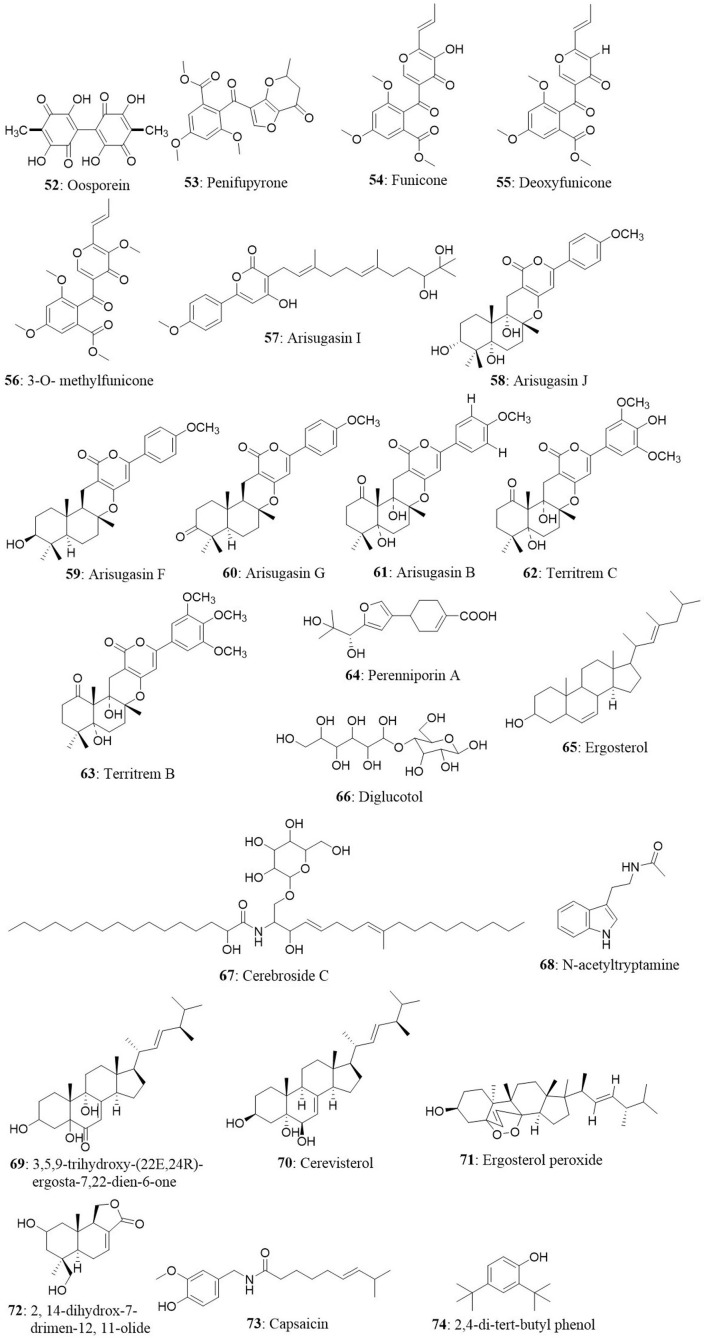



Capsaicin **(73)** along with 2,4-di-tert-butyl phenol **(74)** and a novel compound alternariol-10-methyl ether **(75)** was isolated from *Alternaria alternata* of *Capsicum annuum*. Alternariol-10-methyl ether displayed considerable antiproliferative activity against a panel of human cancer cell lines (HL-60, A431, A549, PC-3, HeLa, MIA PaCa-2, and T47D). Formation of apoptotic bodies, loss of mitochondrial membrane potential and fragmentation of genomic DNA are some of the morphological and electrochemical changes that occur in the cell undergoing to apoptosis (Rakesh et al., 2014; Roopashree et al., 2015). On treatment of HL-60 with alternariol-10-methyl ether, change in the nuclear morphology and loss of mitochondrial membrane potential was observed (Devari et al., 2014).

In another study, endophytes were isolated from different parts of hazelnut (*Corylus avellana* L.) and the isolate was identified as *Phomopsis amygdali*. Further analysis revealed the presence of two metabolites namely (S)-4-butoxy-6-((S)-1-hydroxypentyl)-5,6-dihydro-2H-pyran-2-one **(76)** and pestalotin **(77)**. Both compounds were tested for cytotoxicity against MDA-MB-231, PC-3, HT-29, and HEK293 cell lines. Compound 76 presented good activity against MDA-MB-231 (IC_50_: 24.26 μg/mL) and moderate activity against PC-3 and HT-29 (132.23 and 82.18 μg/mL, respectively). On the other hand, pestalotin showed good cytotoxic activity against the PC-3, HT-29, and MDA-MB-231 cell lines with IC_50_ of 20.54, 16.69, and 41.70 μg/mL respectively (Akay et al., 2014). In the same study, authors investigated the presence of gene region of taxadiene synthase (a key enzyme in taxol biosynthesis) in the *Phomopsis amygdali* isolate using PCR. The results demonstrated the absence of Ts gene region indicating that isolate may not produce taxol.

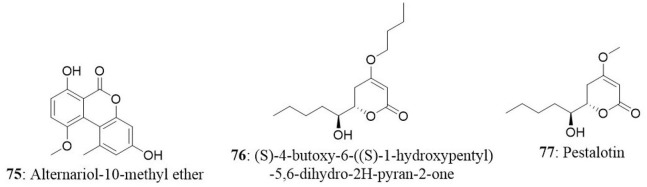



The *Aspergillus* sp. fungus was isolated from medicinal plant *Gloriosa superba*. The bioassay-guided fractionation resulted in an isolation of a novel compound colchatetralene **(78)** along with the three known compounds namely 5-(hydroxymethyl)furan-2 carbaldehyde **(79)**, 4-hydroxy-phthalic acid-dimethyl ester **(80)**, and ergosterol **(65)**. All four compounds were subjected to their cytotoxic effect against A549, HEp2, MCF7, OVCAR5, THP1, and CV1 cell lines. Colchatetralene was found to be effective against THP-1 and MCF7 with an IC_50_ value of 30 and 50 μg/mL, respectively (Budhiraja et al., 2013). In another report, two strains of the endophytic fungi *Penicillium melinii* Yuan-25 and *Penicillium janthinellum* Yuan-27 were isolated from the roots of *Panax ginseng* at Changchun, Jilin Province, China. Bioactivity oriented isolation from the Yuan-25 culture yielded a new benzaldehyde derivative, ginsenocin **(81)**, along with five known compounds, methyl 2,4-dihydroxy 3,5,6-trimethylbenzoate **(82)**, 3,4,5-trimethyl-1,2-benzenediol **(83)**, penicillic acid **(84)**, mannitol **(85)**, and ergosterol peroxide **(71)**. On the other hand, brefeldin A **(11)** was isolated as a major constituent from the Yuan-27 culture. The isolated compounds were tested for their cytotoxic potential against six human cancer cell lines (MKN45, LOVO, A549, MDA-MB-435, HepG2, and HL-60). Brefeldin A exhibited the maximum cytotoxicity against the all tested cell lines with IC_50_ values less than 0.12 μg/mL. Ginsenocin and penicillic acid also showed effective cytotoxicity with IC_50_ values ranging from 0.49 to 7.46 μg/mL against tested cancer cells (Zheng et al., 2013).

The endophytic fungus *A. tenuissima* was obtained from the bark of *Erythrophleum fordii* Oliver at Nanning, Guangxi Province, China. Tenuissimasatin **(86)**, a novel isocoumarin, and other 11 known metabolites were isolated from *A. tenuissima*. Coumarin derivatives are known for their anticancer properties and their effect on various cancer models is well-documented (Keerthy et al., 2014; Neelgundmath et al., 2015). Among the 12 compounds (6aR,6bS,7S)-3,6a,7,10-tetra-hydroxy-4,9-dioxo-4,6a,6b,7,8,9 hexahydroperylene (**87)** illustrated good cytotoxic activity toward HCT-8 cells with IC_50_ value of 1.78 μmol/L. Other compounds showed relatively less activity with IC_50_ value more than 10 μM/L (Fang et al., 2012). A novel metabolite, chaetoglobosin X **(88)**, together with three known compounds (erogosterol, ergosterol 5,8-peroside, and 2-methyl-3-hydroxy indole) were isolated from the endophyte *Chaetomium globosum* Kunze. The fungus was isolated from the medicinal plant *Curcuma wenyujin* collected from Zhejiang Province, Wenzhou, China. Chaetoglobosin X exhibited strong cytotoxic activity against H22 cells with an IC_50_ value of 3.125 μg/mL and moderate cytotoxicity against MFC with an IC_50_ value of 6.25 μg/mL (Wang et al., 2012).

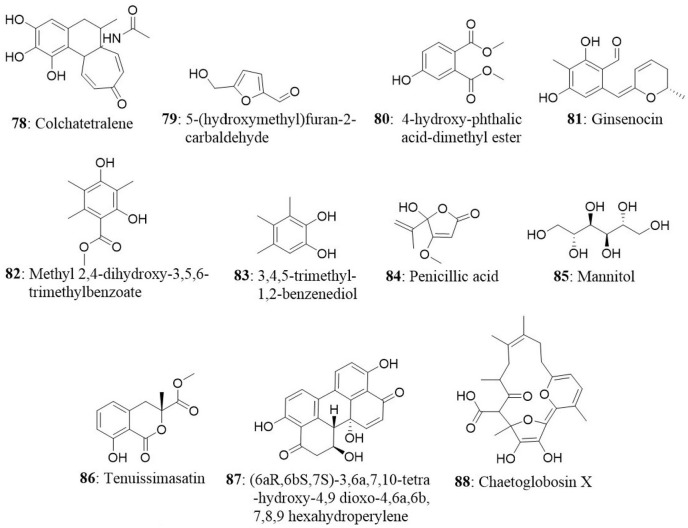



The bioactive metabolites of *Penicillium* sp., isolated from the leaves of *Hopea hainanensis* are monomethylsulochrin **(89)**, rhizoctonic acid **(90)**, asperfumoid **(91)**, physcion, 7,8-dimethyl-isoalloxazine, and 3,5-dichloro-p-anisic acid **(92)**. All the six compounds were evaluated for cytotoxicity against KB and HepG2 cells. The compounds 89, 90, 91, and 92 exhibited good cytotoxicity with IC_50_ values of 30, 20, 20, and 5 μg/mL for KB cells; 30, 25, 15, and 10 μg/mL for HepG2 cells respectively (Wang et al., 2008). A novel chlorinated azaphilone derivative named chaetomugilin D **(93)**, together with three known metabolites, chaetomugilin A **(94)**, chaetoglobosin A **(95)** and C **(96)**, were acquired from *C. globosum*. The endophytic fungus was isolated from the leaves of *G. biloba* in Linyi, Shandong province, China. All the isolated compounds displayed significant growth inhibitory activity against brine shrimp (*Artemia salina*) and *Mucor miehei* (Qin et al., 2009).

The novel alkaloids chaetoglobosin V **(97)** and W **(98)**, together with six known congeners were obtained from *C. globosum* isolated from *Artemisia annua* at Nanjing, Jiangsu Province, China. Chaetoglobosin V and W displayed moderate cytotoxic activity against MCF7 and HepG2 cell lines with IC_50_ values of 52.76 and 51.04 μM, respectively. The cytotoxicity of chaetoglobosin V and W was also assessed against KB, K562, MCF7, and HepG2 cells. Chaetoglobosin V displayed the IC_50_ value of 23.53 and 27.86 μg/mL in KB and MCF7 cells whereas chaetoglobosin W showed 21.17 and 27.87 μg/mL against the same cells, respectively. However, both the compounds did not show cytotoxicity in K562 cells up to 30 μg/mL (Zhang J. et al., 2010).

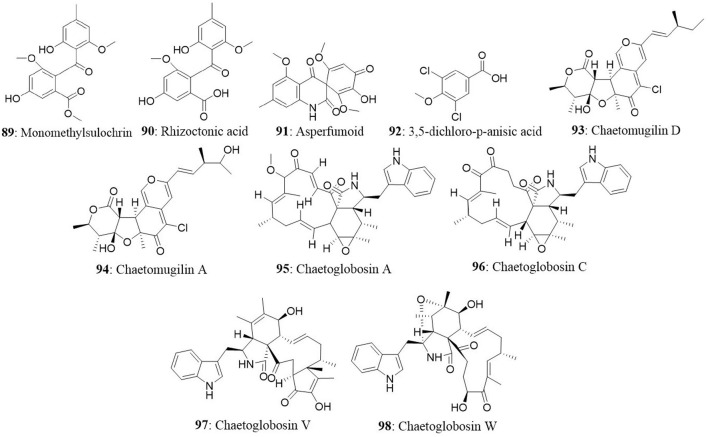



An endophytic fungus, *Botryotinia fuckeliana* isolated from *Ajuga decumbens* Thunb, produced two new (1-keto-4a,15-epoxyeudesm-11-ol and ent-4(15)-eudesmen-5,6-ol-1-one) and four known (ent-4(15)-eudesmen-11-ol-1-one, phenochalasin B, [12]-cytochalasin, [1,3] dioxacyclotridecino) metabolites. Among the isolated compounds, phenochalasin B **(99)** and [12]-cytochalasin **(100)** exhibited significant inhibitory activity with IC_50_ values of 7.43 and 0.88 μM for SMMC-7721 cells; 0.83 and 0.66 μM for A549 cells; 2.22 and 0.062 μM for HepG2 cells; and 0.10 and 0.59 μM for MCF7 cells respectively. The cytotoxic effect of phenochalasin B was significant on A549, HepG2, and MCF7 cancer cells compared with the SMMC-7721 cells (Lin et al., 2015).

Rohitukine **(101)** is a chromane alkaloid with anticancer, anti-inflammatory, and immunomodulatory properties. The endophytic fungus, *Fusarium proliferatum* was isolated from the inner bark of the tree *Dysoxylum binectariferum* Hook.f growing in Central Western Ghats of Karnataka, India. The yield of rohitukine was 186 μg/100 g dry mycelial weight. The methanolic extract of *F. proliferatum* was found to be cytotoxic against the HCT-116 and MCF7 cell lines with an IC_50_ value of 10 μg/mL for both the cell lines and the IC_50_ value of positive control (Camptothecin) was found to be 1 μg/mL (Mohana Kumara et al., 2012). The culture of *F. oxysporum* was isolated from the bark of *Cinnamomum kanehirae* from Jiaoban mountain, Taiwan province. The fungal metabolite analysis revealed the presence of two new compounds namely oxysporidinone analog **(102)**, 3-hydroxyl-2-piperidinone derivative **(103)**, along with seven known compounds namely (–)-4,6′-anhydrooxysporidinone **(104)**, (+)-fusarinolic acid **(105)**, gibepyrone D **(106)**, beauvercin **(107)**, cerevisterol **(70)**, fusaruside **(108)**, and (2S,2′R,3R,3′E,4E,8E)-1-O-D-glucopyranosyl-2-N-(2′-hydroxy-3′-octadecenoyl)-3-hydroxy-9-methyl-4,8-sphingadienine **(109)**. Subsequently, all the compounds were evaluated for cytotoxicity against the PC-3, Panc-1, and A549. The results showed that beauvericin is moderately cytotoxic against PC-3, Panc-1, and A549 with IC_50_ values of 49.5, 47.2, and 10.4 μM. The other compounds displayed weak cytotoxicity for all the tested cell lines (Wang et al., 2011). Endophytes from *Clidemia hirta* (a perennial shrub) were isolated, and analyses of the endophytic *Cryptosporiopsis* sp., led to the isolation of three bioactive molecules, (R)-5-hydroxy-2-methylchroman-4-one **(110)**, and two novel compounds 1-(2,6-dihydroxyphenyl)pentan-1-one **(111)**, and (Z)-1-(2-(2-butyryl-3-hydroxyphenoxy)-6-hydroxyphenyl)-3-hydroxy but-2-en-1-one **(112)**. Compound 110 was presented as a lead cytotoxic agent against the HL-60 with IC_50_ of 4 μg/mL. This compound significantly induced arrest of cell cycle in G2 phase in HL-60 cells (Zilla et al., 2013). Inflammation is called as double-edged sword because deregulation in inflammatory signaling pathways may lead to cancer (Srinivas et al., 2015). Therefore, inflammation and cancer are closely entangled. In the next study, the endophytic fungus Cs-c2 (*Phomopsis* sp.) was isolated from healthy adult leaves of *Senna spectabilis* (Fabaceae). Thereafter, secondary metabolites were extracted and evaluated the anti-inflammatory activities. The chemical investigation of the fungus revealed the presence of a new compound (2-hydroxy-alternariol) together with six known compounds (cytochalasins J and H, 5'-epialtenuene, alternariol monomethyl ether, alternariol, and cytosporone C). Cytochalasin J and H, and alternariol exhibited good anti-inflammatory activity (Chapla et al., 2014). In another study, hydroxy-2-methoxy-5-methylpyridin-2(1H)-one (α-pyridone derivative), 3-hydroxy-N-(1-hydroxy-3-methylpentan-2-yl)-5-oxohexanamide (a ceramide derivative), and 3-hydroxy-N-(1-hydroxy-4-methylpentan-2-yl)-5-oxohexanamide (a new natural compound) along with 15 known compounds were isolated from an endophytic fungus *Botryosphaeria dothidea* KJ-1. The fungus was collected from the symptomless tissue of stem bark of *Melia azedarach* L. (white cedar) at Yangling, Shaanxi province, China. The results presented that the new compounds did not induce a significant cytotoxic effect on HCT116 cells up to 100 μM. However, the known compounds exhibited cytotoxic effect with IC_50_ values ranging between 3.13 and 73.4 μM (Xiao et al., 2014).

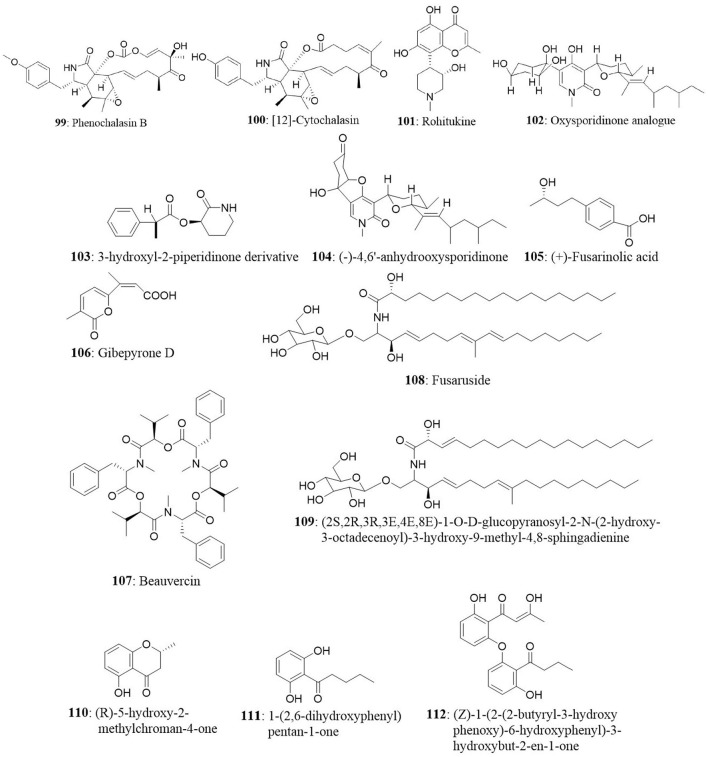



## Cytotoxic metabolites of endophytic fungi from mangrove plants

Mangrove endophytic fungi are the second largest ecological group of marine fungi and are potential sources for isolation of new bioactive compounds (Kalaiselvam, 2015). They have attracted researchers due to their importance in ecology (Calcul et al., 2013). Presently, only a small number of mangrove fungi have been studied. Fungal endophytic associations with mangrove allow them to successfully compete with saprobiotic fungi for decomposition of their senescent parts (Elavarasi et al., 2012). Mangrove-associated fungi provide a wide array of bioactive secondary metabolites with unique structures including alkaloids, benzopyranones, chinones, flavonoids, phenolic acids, quinones, steroids, terpenoids, tetralones, and xanthones (Huang et al., 2011). In the following section, natural compounds isolated from endophytic fungi from mangrove plants have been discussed.

Seven fungal strains were isolated from the leaves of *Rhizophora mucronata* and *Avicennia officinalis*. One of the isolated endophytic fungi obtained was identified as *Irpex hynoides*. The fungal extract showed potent cytotoxic activity against HEp2 cell line and the bioactive metabolite in the extract was predicted to be tetradecane (Bhimba et al., 2011). The endophytic fungus Zh6-B1 from *Sonneratia apetala* produces two new metabolites namely 3R,5R-Sonnerlactone **(113)**, 3R,5S-Sonnerlactone **(114)**, and two known compounds namely 3,4-dihydro-4,8-dihydroxy-7-(2-hydroxyethyl)-6-methoxy-1(2H)-naphthalen-1-one and 10-norparvulenone. The antiproliferative activity of new compounds was evaluated against multi-drug resistant human oral floor carcinoma (KV/MDR) cells. Both the compounds (113 and 114) inhibited KV/MDR cell growth by 42.4 and 41.6% at 100 μM, respectively (Li et al., 2010). The endophyte *F.oxysporum* from *Rhizophora annamalayan* was identified as taxol-producing fungus. The amount of taxol produced by the fungus was found to be 172.3 μg/L (Elavarasi et al., 2012). Anthracenedione derivatives were extracted from the secondary metabolites of mangrove endophytic fungi *Halorosellinia* sp. (No. 1403) and *Guignardia* sp. (No. 4382) isolated from Kandelia woody tissue from the South China Sea. Of the 14 compounds investigated, 1-hydroxy-3-methylanthracene-9,10-dione **(115)** induced apoptosis in KB and KBv200 cells and displayed cytotoxicity with IC_50_ values of 3.17 and 3.21 μM respectively (Zhang J. Y. et al., 2010). The endophyte *Phomopsis* sp. ZSU-H76 produced a new polyketide, 2-(7′-hydroxyoxooctyl)-3-hydroxy-5-methoxybenzeneacetic acid ethyl ester **(116)** and three known compounds (Dothiorelone A, B, and C). This endophyte was isolated from the stem of *Excoecaria agallocha* in Dong Zai, Hainan, China. The polyketide exhibited cytotoxicity toward HEp2 and HepG2 cell lines with IC_50_ values of 25 and 30 μg/mL, respectively (Huang et al., 2009). The endophytic fungus *Paecilomyces* sp. was isolated from the bark of an unidentified mangrove from the Taiwan Strait and it was found to produce two new depsidone-type metabolites named paeciloxocin A **(117)** and B **(118)**. Paeciloxocin A exhibited significant cytotoxicity against HepG2 cell line with the IC_50_ value of 1 μg/mL, whereas paeciloxocin B showed less cytotoxic activity with an IC_50_ value of 65 μg/mL (Wen et al., 2010).

The culture of *Penicillium expansum* was isolated from roots of the mangrove plant *Excoecaria agallocha* (Euphorbiaceae). The fungus yielded four new polyphenols [expansol A **(119)**, B **(120)**, (S)-(+)-11-dehydrosydonic acid **(121)**, and (7S,11S)-(+)-12-acetoxysydonic acid **(122)**] and two known compounds [(*S*)-(+)-sydonic acid and diorcinol]. Expansol A and B contain phenolic bisabolane sesquiterpenoid as well as diphenyl ether units in their structure. All the isolated compounds were evaluated for cytotoxicity against the A549 and HL-60 cell lines. Expansol A presented moderate antiproliferative activity against the HL-60 cell line with the IC_50_ value of 15.7 μM, whereas expansol B remarkably suppressed the proliferation of both cell lines with IC_50_ values of 1.9 and 5.4 μM, respectively (Lu et al., 2010).

Mangrove-derived endophytic fungus *Fusarium* sp. was isolated from the fresh stems of *Kandelia candel* (Rhizophoraceae) and metabolite analysis revealed the presence of a new isoflavone [5-O-methyl-2′-methoxy-3′-methylalpinumisoflavone **(123)**] together with four known compounds [6-methoxy-5,7,4′-trihydroxyisoflavone, 6-methoxy-7,4′-dihydroxyisoflavone, (+)-marmesin and 4-methylbenzoic acid carboxymethyl ester]. Cytotoxic activity studies against the HEp2 and HepG2 cell lines revealed that compound 123 inhibit cell proliferation significantly with IC_50_ values of 4 and 11 μM, respectively (Huang et al., 2010).

Three new bianthraquinone derivatives, alterporriol K **(124)**, L **(125)**, and M **(126)**, together with six known compounds (physcion, marcrospin, dactylariol, tetrahydroaltersolanol B, alternariol, and alternariol methyl ether) were isolated from *Alternaria* sp. ZJ9-6B from *Aegiceras corniculatum* collected from the South China Sea. In preliminary studies, Alterporriol K and L imparted moderate growth inhibitory activity against MDA-MB-435 and MCF7 cells with IC_50_ values ranging from 13.1 to 29.1 μM (Huang et al., 2011). An ergochrome class mycotoxin, secalonic acid D **(127)** was isolated from the endophytic fungus No. ZSU44 and evaluated for its anticancer activity against HL60 and K562 cells. The results showed strong cytotoxicity towards HL60 and K562 cells with IC_50_ values of 0.38 and 0.43 μM, respectively. Furthermore, apoptosis inducing effect of secalonic acid D was confirmed by annexin V-FITC/PI staining. Activation of executioner caspase and PARP cleavage are the crucial events in the cells undergoing apoptosis (Ashwini et al., 2015; Baburajeev et al., 2015). Secalonic acid D induced the activation of caspase-3 and cleavage of PARP in HL60 and K562 cells (Zhang J. Y. et al., 2009). The endophytic fungus *Phomopsis* sp. was isolated from *Excoecaria agallocha* (Euphorbiaceae) in Dong Zai, Hainan, China. The secondary metabolite analysis revealed the presence of three unique compounds namely phomopsis-H76 A, B, and C **(128-130)**. These metabolites did not show considerable cytotoxic activity against KB, KBv200, and MCF7 cell lines and IC_50_ values were over 50 μM (Yang et al., 2010).

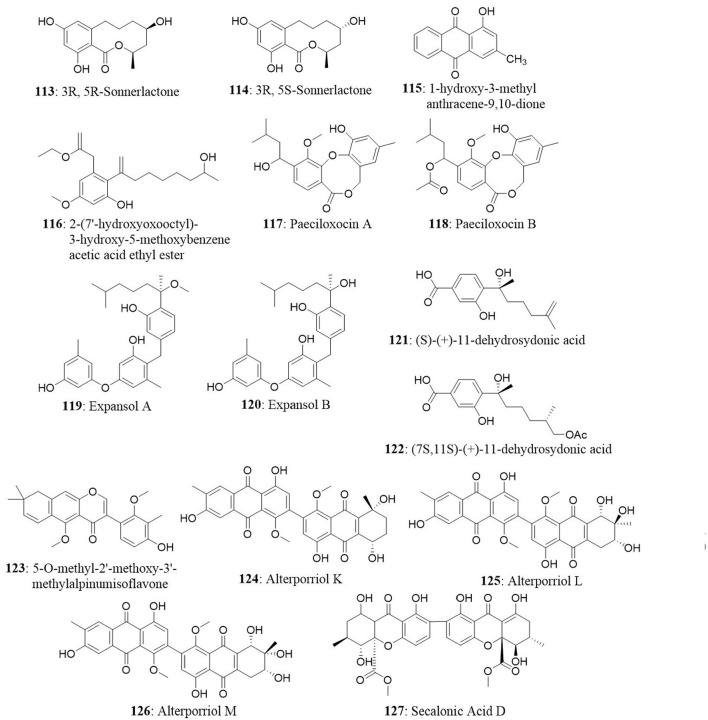



Three new sesquiterpenes, merulin A **(131)**, B and C **(132)** were produced by the endophytic fungus XG8D (Basidiomycete) isolated from the mangrove plant *Xylocarpus granatum* (Meliaceae). Merulin A and C exhibited good cytotoxic activity against human breast and colon cancer cell lines with IC_50_ values of 4.98 and 1.57 μg/mL for BT474; 4.84 and 4.11 μg/mL for SW620, respectively. Doxorubicin was used as vehicle control with IC_50_ values of 0.09 and 0.53 μg/mL against SW-620 and BT-474 cell lines, respectively. (Chokpaiboon et al., 2010). A new pyrrolyl 4-quinolinone alkaloid, penicinoline **(133)**, was isolated from *Penicillium* sp., from the bark of the mangrove *Acanthus ilicifolius* (Acanthaceae) collected from the South China Sea. Intramolecular dehydration of the compound yielded an unexpected lactam derivative **(134)**, which was confirmed by single-crystal X-ray analysis. Penicinoline showed potent cytotoxicity with IC_50_ values of 0.57 and 6.5 μg/mL toward the 95-D and HepG2 cell lines, respectively. However, penicinoline was inactive against HeLa, KB, KBv200, and HEp2 cell lines at the concentration of 100 μg/mL (Shao et al., 2010).

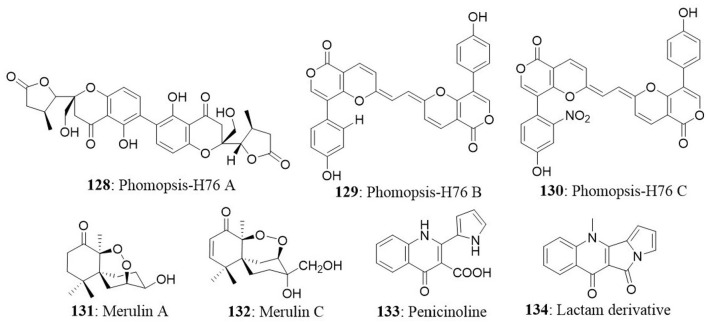



The endophytic *Pestalotiopsis* sp. was obtained from the leaves of *Rhizophora mucronata* from Dong Zhai Gang, Mangrove Garden on Hainan Island, China. The endophyte produced five novel cytosporones J-N **(135-139)**, five new pestalasins A-E **(140-144)**, and a new alkaloid pestalotiopsoid A **(145)**. All the isolated compounds were investigated for their cytotoxicity against L5178Y, HeLa, and PC12 cells. All the compounds were found to be inactive at a concentration of 10 μg/mL (Xu et al., 2009b). The *Pestalotiopsis* sp. produced six new pestalotiopsones A–F (chromones) **(146-151)** and a new compound 7-hydroxy-2-(2-hydroxypropyl)-5-methylchromone. The fungus was isolated from the leaves of *Rhizophora mucronata*. Among the isolates, only pestalotiopsone F exhibited moderate cytotoxicity against the murine cancer cell line (L5178Y) with an EC_50_ value of 8.93 μg/mL (Xu et al., 2009a).

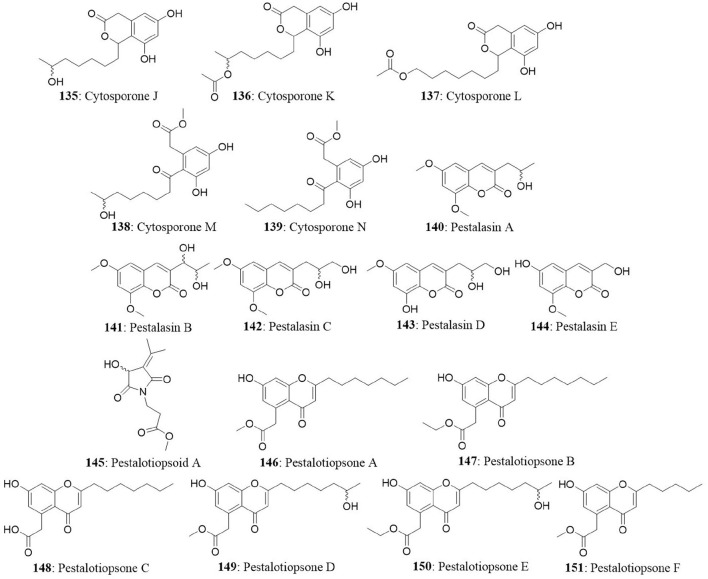



Five highly oxygenated chromones named rhytidchromones A-E **(152-156)** were isolated from the endophytic fungus *Rhytidhysteron rufulum* BG2-Y. The fungal strain was isolated from the healthy leaves of *Bruguiera gymnorrhiza* collected from Pak Nam Pran, Prachuab Kiri Khan Province, Thailand. Isolated compounds were tested for their cytotoxicity on a panel of cancer cell lines (MCF7, HepG2, Kato-3, and CaSki). All the compounds excluding rhytidchromone D, displayed cytotoxicity toward Kato-3 cell lines with IC_50_ values ranging from 16.0 to 23.3 μM, while rhytidchromones A and C were active against MCF7 cells with IC_50_ values of 19.3 and 17.7 μM, respectively (Chokpaiboon et al., 2016).

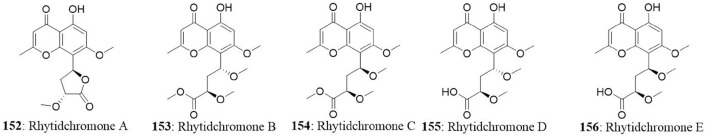



## Endophytic fungal metabolites from marine habitats

Marine fungi comprise of obligate and facultative types; and classically they are defined as a form of ecological, but not a taxonomic group of fungi (Elsebai et al., 2014). The growth and sporulation of obligate marine fungi occur exclusively in marine environment. In case of facultative marine fungi, the growth, and sporulation in the marine habitat are dependent on the physiological adaptations (Raghukumar, 2008). All marine habitats such as marine plants (mangrove plants, algae, seagrasses, and driftwood), marine vertebrates and invertebrates (corals, bivalves, sponges, and crustaceans), and abiotic factors (soil) can host fungal strains. Many reviews have not focused significantly on bioactive natural compounds from endophytic fungi of marine origin. The fungal diversity in marine habitats has been underestimated because of their propensity to form aggregates (Rateb and Ebel, 2011). Sponges and algae contribute majorly as sources of fungi in marine habitats. A thorough investigation of algae and sponges is essential to explore the fungal communities associated with them. The marine environment has extreme conditions like high salinity, more amount of metals in hydrothermal vents, elevated hydrostatic pressure, and low temperatures in the deep sea (Raghukumar, 2008). The organisms that live and thrive in the adverse and extreme conditions can be expected to produce metabolites that are unique and might be of therapeutic interest. Therefore, it is essential to search for exotic and unique metabolite-producing organisms in these unusual environments. Marine-derived fungi are a prolific source of new bioactive compounds with a high degree of structural diversity and equally interesting pharmacological activities, namely, anticancer, antibacterial, antifungal, and antiviral activities, as well as involvement in specific enzyme inhibition activities (Bugni et al., 2004). The bioactive natural compounds from marine fungi have been investigated to smaller extents relatively with their terrestrial counterparts (Vita-Marques et al., 2008; Gamal-Eldeen et al., 2009). Several research groups have been involved in the isolation of marine microbes, analysis of their secondary metabolites and evaluation of possible pharmacological properties.

The *Talaromyces* sp. was isolated from seaweed in Kasai Rinkai Park, Tokyo, Japan. Two novel azaphilones, kasanosins A **(157)** and B **(158)** were isolated from cultures of *Talaromyces* sp. These compounds are evaluated for their ability to inhibit mammalian DNA polymerases, the enzyme responsible for DNA replication. The results demonstrated that kasanosin A and B selectively inhibit eukaryotic DNA polymerases β and γ. Kasanosin A was more active than kasanosin B, with IC_50_ values of 27.3 μM (rat DNA polymerase β) and 35 μM (human DNA polymerase λ), respectively (Kimura et al., 2008). Alterporriol L **(52)** is a new bianthraquinone derivative isolated from a marine fungus *Alternaria* sp. ZJ9-6B. The fungus was isolated from the plant *Aegiceras corniculatum* in the South China Sea. The cytotoxic effect of alterporriol L was investigated against MCF7 and MDA-MB-435 cells. The IC_50_ value was found to be 20.04 μM for MCF7 and 13.11 μM for MDA-MB-435 (Huang et al., 2012). *Penicillium* sp. was isolated from *Limonium tubiflorum* and the chemical investigation of fungal extract revealed the presence of four new compounds namely penilactone **(159)**, 10, 11-epoxycurvularin **(160)**, neobulgarone G **(161)**, and sulfimarin **(162)** along with 12 known metabolites. NF-κB is a proinflammatory transcription factor that is persistently activated in several cancers (Neelgundmath et al., 2015; Nirvanappa et al., 2016). The new compounds (159-162) did not show pronounced inhibition against TNFα-induced NF-κB activity in K562 cells (Aly et al., 2011). The fungus *Aspergillus versicolor* from the marine sponge *Petrosia* sp. yielded an aromatic polyketide derivative (2,4-Dihydroxy-6-((R)-4-hydroxy-2-oxopentyl)-3-methylbenzaldehyde), two xanthones (sterigmatocystin and dihydrosterigmatocystin) and five anthraquinones (averantin, methylaverantin, averufin, nidurufin, and versiconol). Among the eight isolates, sterigmatocystin **(163)**, averantin **(164)**, methylaverantin **(165)**, and nidurufin **(166)** exhibited significant cytotoxicity against A549, SK-OV-3, SK-MEL-2, XF-498, HCT-15 with IC_50_ values ranging between 0.41 and 4.61 μg/mL (Lee et al., 2010).

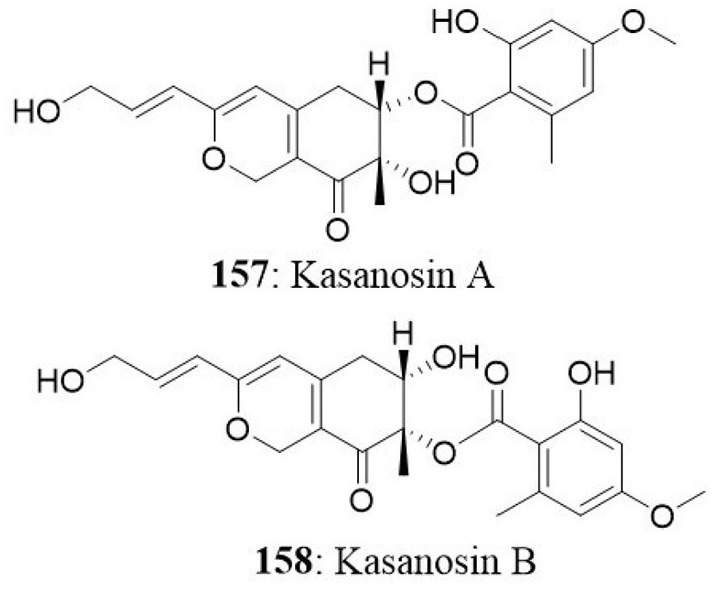

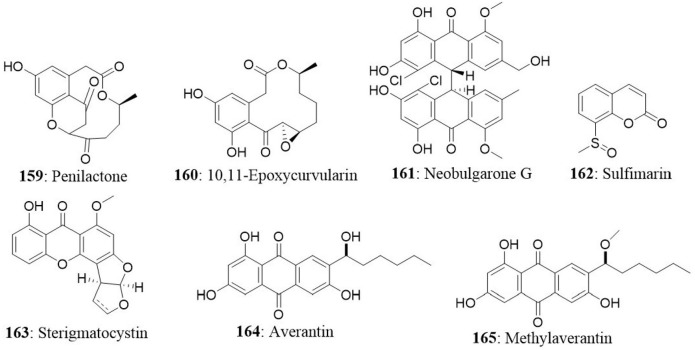

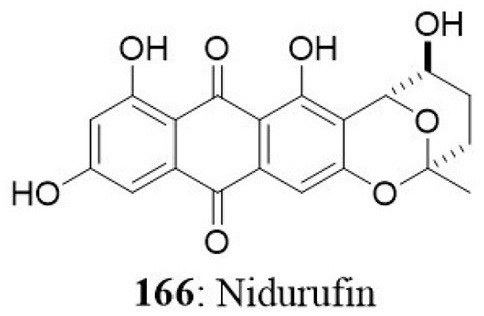







The endophytic fungus *Chaetomium globosum* (QEN-14) was isolated from the marine green alga *Ulva pertusa* (Ulvaceae) collected from Qingdao coastline, China. The fungus yielded seven new cytochalasan derivatives (cytoglobosins A-G) together with two known compounds (isochaetoglobosin D and chaetoglobosin F_ex_). New isolates were evaluated for their cytotoxicity against P388, A549, and KB cancer cell lines. The results indicated that cytoglobosins C **(167)** and D **(168)** as potent anticancer agents towards A549 cells with IC_50_ values of 2.26 and 2.55 μM, respectively (Cui et al., 2010b). The other compounds did not show significant cytotoxic effect up to 10 μM.

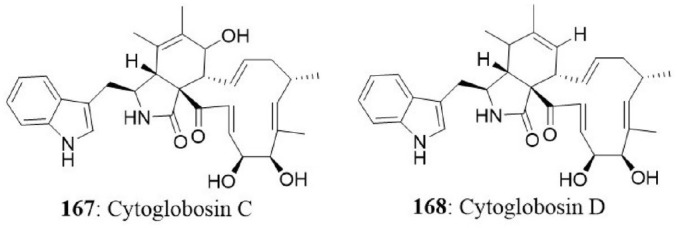



Two prenylated polyketides, epoxyphomalin A **(169)** and B **(170)** were obtained from the facultative marine fungus *Phoma* sp. The fungus *Phoma* sp. was collected from the marine sponge *Ectyplasia perox* at the Caribbean Sea, Dominica. The cytotoxic effects of compounds 169 and 170 were evaluated using a monolayer cell survival and proliferation assay on a 36 human tumor cell lines. Epoxyphomalin A displayed considerable *in vitro* tumor cell selectivity towards 12 of the 36 tested tumor cell lines. The IC_50_ values were ranged from 0.01 to 0.038 μg/mL (Mohamed et al., 2009). The fungus *Curvularia* sp. was isolated from the red alga *Acanthophora spicifera*. Chemical investigations of the fungal isolate yielded eight compounds including apralactone A, antipodes of curvularin macrolides and a dimeric curvularin. A modified propidium iodide monolayer assay was used to determine the cytotoxic activity of the isolated compounds against human tumor cell lines. Apralactone A **(171)** (14-membered phenylacetic acid macrolactone) showed considerable dose-dependent cytotoxicity with a mean IC_50_ value of 9.87 μM. The other compound, (+)-(10E,15R)-10,11-dehydrocurvularin **(172)**, also showed dose-dependent cytotoxicity with a mean IC_50_ value of 1.25 μM (Greve et al., 2008, 2010).

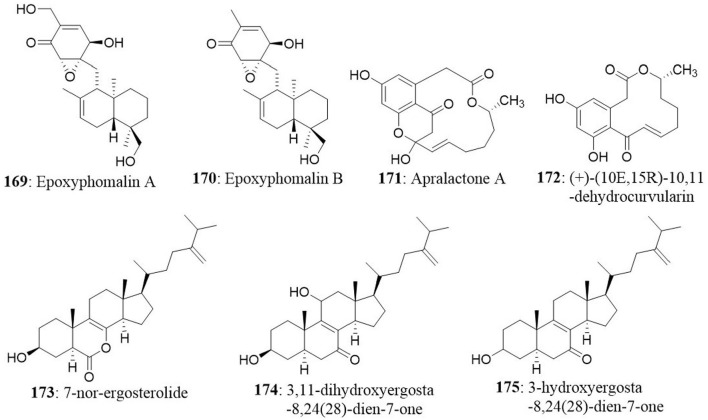



Chemical investigation of *Aspergillus ochraceus* isolated from the marine brown alga *Sargassum kjellmanianum* revealed the production of 7-nor-ergosterolide **(173)** and two new steroidal derivatives [3β,11α-dihydroxyergosta-8,24(28)-dien-7-one **(174)** and 3β-hydroxyergosta- 8,24(28)-dien-7-one **(175)**] and nine known related steroids. Compounds 173, 174, and 175 were tested for their cytotoxic activities against NCI-H460, SMMC-7721, SW1990, DU145, HepG2, HeLa, and MCF7 cancer cell lines. The 7-nor-ergosterolide showed potent, selective cytotoxic activity against the NCI-H460, SMMC-7721, and SW1990 cells with IC_50_ values of 5, 7 and 28 μg/mL, respectively. On the other hand, 3β,11α-dihydroxyergosta-8,24(28)-dien-7-one weakly inhibited the growth of the SMMC-7721 cell line with the IC_50_ value of 28 μg/mL (Cui et al., 2010a).

## Future challenges with endophytes

Endophytic fungi offer new hope and possibilities for the production of novel bioactive compounds, which is challenged due to low yield, lack of information on precise fungi-plant biochemical interaction, scale-up issues, and growth in the axenic state. There is a constant need to manifest these natural secondary metabolites *in vitro*, which despite being accomplished, has shown limited success in commercial production. There is a weak understanding of interactions of endophytic fungal organisms with other associated microbes. The use of plant tissue culture techniques to obtain higher yields has not been successful due to genetic instability in culture, slow growth of fungi, the formation of cell aggregates, and sensitivity to shearing (Charlwood and Rhodes, 1990). Additionally, the maintenance of tissue cultures is comparatively more expensive and time-consuming than fermentation technologies. Nevertheless, fungal fermentation provides the platform for the potent and sustainable production of bioactive compounds. Microbial fermentation offers certain advantages such as a simple fungal cell medium, low cost, faster growth rate of fungi with minimal risk of contamination, and *in vitro* optimized fermentation conditions that facilitate the production of secondary metabolites but fails to meet the requirements for sustained production, as observed *in vivo* in the host apoplast region. Studies examining genetic aspects to trigger specific genes for overproduction of target metabolites could also be beneficial. Another important hurdle is the difficulties associated with using a combinatorial chemical synthesis approach to generate complex metabolites of the bioactive compounds. These shortcomings may overcome by the use of inducers/elicitors of the specific biochemical pathway that can ultimately increase the yield. The complex structure of endophytic metabolites is a hurdle for use of the combinatorial chemical synthesis approach. Alternatively, the limitations may overcome by the use of signaling molecules and inexpensive precursor feeding in growth medium, which may act as efficient triggers of the biochemical pathway to achieve an elevated yield. Moreover, the presence of several unnecessary metabolites can interfere with the enzymatic activity in the growth medium. The negative feedback mechanism is a major aspect that must be critically investigated to achieve increase in yield with the fungal fermentation strategy (Zhao et al., 2010a). Endophytic studies employing genetic engineering techniques, transformation studies, gene cluster amplification for metabolite overproduction, mutagenesis, medium manipulation, culture condition optimization, and elicitor addition should help to increase the yield of the desired secondary metabolites (Kusari and Spiteller, 2012; Kusari et al., 2012; Venugopalan and Srivastava, 2015).

## Conclusion

Endophytes have been emerged as privileged sources of bioactive compounds, since after the discovery of taxol from *T. andreanae*. The secondary metabolites of endophytes have been used substantially for the sustainable production of therapeutically important compounds. The limited availability of bioactive principles in plant sources could be surpassed by exploiting the chemical entities in the endophytes. With the unfolding knowledge of the anticancer properties of endophytes and current technological advancements in “omics” and fermentation, endophytic fungi may play a lead role in providing a constant supply of chemotherapeutics with high target specificity and minimal side effects at an affordable cost. The attempts in genetic manipulations, overexpressing of the genes that are essential for the production of the specific bioactive metabolite, and other optimization strategies may contribute to the strain improvement and make endophytes as potential drug sources for the future. Therefore, exploring and exploiting of metabolites from endophytes in terrestrial, mangrove, and marine habitats may provide an excellent avenue for the discovery of drug candidates against deadly human diseases.

## Author contributions

All authors listed have made a substantial, direct and intellectual contribution to the work, and approved it for publication.

### Conflict of interest statement

The authors declare that the research was conducted in the absence of any commercial or financial relationships that could be construed as a potential conflict of interest.
